# Phylogeny of *Leontopodium* (Asteraceae) in China—with a reference to plastid genome and nuclear ribosomal DNA

**DOI:** 10.3389/fpls.2023.1163065

**Published:** 2023-07-31

**Authors:** Xue-Min Xu, Zhen Wei, Jun-Zhe Sun, Qing-Fei Zhao, Yang Lu, Zhen-Long Wang, Shi-Xin Zhu

**Affiliations:** School of Life Sciences, Zhengzhou University, Zhengzhou, China

**Keywords:** *Leontopodium*, chloroplast genome, genome structure, nuclear ribosomal DNA, phylogenetic analysis

## Abstract

The infrageneric taxonomy system, species delimitation, and interspecies systematic relationships of *Leontopodium* remain controversial and complex. However, only a few studies have focused on the molecular phylogeny of this genus. In this study, the characteristics of 43 chloroplast genomes of *Leontopodium* and its closely related genera were analyzed. Phylogenetic relationships were inferred based on chloroplast genomes and nuclear ribosomal DNA (nrDNA). Finally, together with the morphological characteristics, the relationships within *Leontopodium* were identified and discussed. The results showed that the chloroplast genomes of *Filago*, *Gamochaeta*, and *Leontopodium* were well-conserved in terms of gene number, gene order, and GC content. The most remarkable differences among the three genera were the length of the complete chloroplast genome, large single-copy region, small single-copy region, and inverted repeat region. In addition, the chloroplast genome structure of *Leontopodium* exhibited high consistency and was obviously different from that of *Filago* and *Gamochaeta* in some regions, such as *matk*, *trnK (UUU)-rps16*, *petN-psbM*, and *trnE (UUC)-rpoB*. All the phylogenetic trees indicated that *Leontopodium* was monophyletic. Except for the subgeneric level, our molecular phylogenetic results were inconsistent with the previous taxonomic system, which was based on morphological characteristics. Nevertheless, we found that the characteristics of the leaf base, stem types, and carpopodium base were phylogenetically correlated and may have potential value in the taxonomic study of *Leontopodium*. In the phylogenetic trees inferred using complete chloroplast genomes, the subgen. *Leontopodium* was divided into two clades (Clades 1 and 2), with most species in Clade 1 having herbaceous stems, amplexicaul, or sheathed leaves, and constricted carpopodium; most species in Clade 2 had woody stems, not amplexicaul and sheathed leaves, and not constricted carpopodium.

## 1 Introduction

*Leontopodium* R.Br. ex Cass. (Asteraceae) comprises 58 species that are distributed across Asia and Europe (Bayer et al., 2007; Chen et al., 2011). The main distribution of the genus is in central and eastern Asia, including Russia, Japan, South Korea, Mongolia, China and along the Himalaya to the borders of Afghanistan and Pakistan; two species occur in Europe, *L. alpinum* Cass. and *L. nivale* (Ten.) Huet ex Hand.-Mazz. (Safer et al., 2011). China has the largest number of *Leontopodium* species in the world, with a total of 37 species, of which 17 are endemic, and the West and Southwest regions of China are the diversity centers of this genus (ca. 20 species) (Lin, 1979; Chen et al., 2011). The genus is mainly characterized by capitula surrounded by bracteal leaves, involucral bracts with membranous margins, central florets that are functionally male, and pappi of bisexual flowers that are usually slightly thicker (Lin, 1979; Chen et al., 2011). Some taxa of this genus, such as *L. artemisiifolium* (Levl.) Beauv., *L. calocephalum* var. *uliginosum* Beauv., and *L. leontopodioides* (Willd.) Beauv. are commonly used as herbal remedies in China because of their antioxidant, anti-inflammatory, anti-rheumatic, and anti-diabetic properties (Lin, 1979; Jiao et al., 1997; Huang and Wu, 2006; Wu et al., 2013).

The phylogenetic position and closely related genera of *Leontopodium* are well defined. Traditionally, *Leontopodium* has been classified as belonging to the tribe Inuleae Cass. (Bentham, 1873; Cassini, 1822; Lin, 1965; Merxmüller et al., 1977; Lin, 1979). Subsequently, tribe Gnaphalieae Cass. ex Lecoq & Juill. was widely accepted based on morphological characteristics and molecular phylogenetic results, with *Leontopodium* belonging to it (Anderberg, 1989; Jansen et al., 1991; Anderberg, 1991a; Anderberg, 1991b; Anderberg, 1991c; Karis, 1993; Bayer and Starr, 1998; Eldenäs et al., 1999; Bayer et al., 2007; Galbany-Casals et al., 2010; Fu et al., 2016; Nie et al., 2016; Smissen et al., 2020). The latest research indicated that Gnaphalieae were one of the larger tribes of Asteraceae with c. 2,100 species in 178 genera, occurring globally across a wide range of temperate habitats (Smissen et al., 2020). Based on *rpl32-trnL*, *trnL* intron, *trnL-trnF*, and the nuclear ribosomal DNA internal transcribed spacer (ITS) and external transcribed spacer (ETS), Galbany-Casals et al. (2010) reported that *Leontopodium* had a close relationship with *Antennaria* Gaertn., *Bombycilaena* (DC.) Smoljan., *Gamochaeta* Wedd., *Evax* Gaertn., *Filago* Loefl., etc.; thus, the “FLAG clade” was proposed (*Filago*, *Leontopodium*, *Antennaria*, and *Gamochaeta* are the largest genera in this clade). In addition, the results of previous molecular phylogenetic studies support the monophyly of *Leontopodium*. Nie et al. (2016) explored the phylogenetic relationships within Gnaphalieae based on abundant samples (a total of 835 terminal accessions representing 80% of the genera, including 27 *Leontopodium* species) and using ITS and ETS sequences; their results supported the “FLAG clade” and indicated that *Leontopodium* was monophyletic. Smissen et al. (2020) suggested a subdivision of Gnaphalieae into two subtribes based on published studies and their new phylogenetic analyses; these were a largely African-endemic Relhaniinae (124 species in 11 genera) and a much enlarged Gnaphaliinae, the latter accounting for more than 90% of the species diversity (c. 2,000 species in 167 genera) and including six clades, Ifloga, Metalasia, Stoebe, HAP, FLAG (*Leontopodium* was in this clade and monophyletic), and Australasian clades.

However, the infrageneric taxonomy system, species delimitation, and interspecies systematic relationships of *Leontopodium* remain controversial and complex. Handel-Mazzetti (1928) accepted 41 *Leontopodium* species and divided the genus into two subgenera (subgen. *Paragnaphalium* Hand.-Mazz. and subgen. *Euleontopodium* Beauv.), and two sections (sect. *Nobilia* (Beauv.) Hand.-Mazz. and sect. *Alpina* Hand.-Mazz.); only one species was included in subgen. *Paragnaphalium* (*L. forrestianum* Hand.-Mazz.). Blöch et al. (2010) treated several of the taxa proposed by Handel-Mazzetti (1928) as synonyms and pointed out that *Leontopodium* may comprise only 30 species. Lin (1965) indicated the existence of about 40 species and 12 natural hybrids of *Leontopodium* in China and divided this genus into two subgenera (subgen. *Paragnaphalium* and subgen. *Leontopodium*), two sections (sect. *Nobilia* and sect. *Leontopodium*), eight subsections, and 12 series. Whereas Chen et al. (2011) revised *Leontopodium* in China and indicated that there were only 37 species in this genus; moreover, they did not set up an infrageneric taxonomy system and did not accept hybrids, because it was somewhat difficult to distinguish them according to the knowledge available at that time. To date, only two studies have focused on the molecular phylogeny of some *Leontopodium* species. Blöch et al. (2010) explored the relationships of 22 *Leontopodium* species using three chloroplast markers (*matK*, *trnL* intron, and *trnL-trnF*) and two nuclear genes (ITS and ETS); their results showed that the Southeast Tibetan monotypic *Sinoleontopodium* (*S. lingianum* Y.L. Chen) fell into *Leontopodium*, and *L. lingianum* (Y.L. Chen) Dickoré, *comb. nov.*, was proposed, to ensure the monophyly of *Leontopodium*. Safer et al. (2011) divided 16 *Leontopodium* species into 10 groups according to the results of Amplified Fragment Length Polymorphism, to discuss the relationships within the genus and to reveal information about its biogeography. Thus, few taxa were included, and few molecular markers have been used in previous phylogenetic studies of *Leontopodium*, which has led to incomplete and indistinct interspecies phylogenetic relationships. Accordingly, additional molecular data and taxa should be used to investigate the interspecies relationships of *Leontopodium*.

Phylogenetic analyses using chloroplast (cp) and nuclear genes are more comprehensive and can be used to explore complex genetic relationships. The cp, which has independent genetic material (mainly maternally inherited), is responsible for photosynthesis and plays important roles in other aspects of plant physiology and development, including the synthesis of various proteins, nucleotides, carbohydrates, and metabolites (Leister, 2003; Xiong et al., 2009; Wicke et al., 2011; Daniell et al., 2016; Tian et al., 2021). Generally, the cp genome has a typical quadripartite circular structure comprising two copies of inverted repeat (IR) regions, a large single-copy (LSC) region, and a small single-copy (SSC) region (Palmer, 1985; Qian et al., 2021). In addition, cp genomes are highly conserved, not only in structure, but also in gene number and composition, usually ranging from 120 to 220 kb and including 120–130 genes (Jansen et al., 2008; Rogalski et al., 2015). Moreover, the evolutionary rate of cp genomes is relatively moderate and lies between those of the nuclear and mitochondrial genomes (Dong et al., 2013). Owing to the lack of recombination, small genome size, and high copy number per cell, complete cp genome sequences have significantly contributed to phylogenetic studies and plant classification (Dong et al., 2012; Twyford and Ness, 2017; Jiang et al., 2020; Pascual-Díaz et al., 2021; Sun et al., 2021; Wang et al., 2021; Zhang X. F. et al., 2021). Mutation hotspot regions and single-sequence repeats can be identified by comparing cp genome sequences and are commonly used as effective molecular markers for species identification and classification, population genetics, and evolutionary studies (Dong et al., 2012). Furthermore, because the variation in genome structure is often considered a type of evolutionary event in general (Jansen and Ruhlman, 2012), the differences in the cp genome structure are often analyzed in great detail among species (Jansen et al., 2006; Liu et al., 2013; Barkalov and Kozyrenko, 2014; Lee et al., 2016; Ross et al., 2016; Ng et al., 2017; Sam et al., 2017; Wei et al., 2017; Wu et al., 2017; Zhang et al., 2017; Yang et al., 2018; Jiang et al., 2020; Sun et al., 2021). Currently, the National Center for Biotechnology Information (NCBI) database includes many complete cp genomes of Asteraceae (approximately 1760) (https://www.ncbi.nlm.nih.gov/). However, to date, only one complete cp genome of *Leontopodium* (*L. leiolepis* Nakai, KM267636) has been published in the NCBI database. Therefore, additional cp genome data must be analyzed to unveil the infrageneric taxonomic system, species delimitation, and interspecies systematic relationships of *Leontopodium.* Compared with genomic data, phylogenetic analysis based on DNA barcodes, such as *trnL-F*, *rbcL*, *matK*, *trnK-matK*, *psbA-trnH*, ITS, and ETS, can solve many taxonomic problems, among which ITS and ETS are important nuclear DNA fragments that are widely used in phylogenetic studies (Kuzmanović et al., 2017; Malekmohammadi et al., 2017; Vicent et al., 2017; Zhou and Zhang, 2017; García et al., 2018; Asanuma et al., 2019; Hussain et al., 2019; Pirani et al., 2020; Tan et al., 2020; Zhou et al., 2020; Hashim et al., 2021). ITS and ETS have fast evolutionary rates and high interspecies variation, which are reportedly effective in discriminating closely related species with relatively recent divergences (Li et al., 2011). In addition, incongruent genetic relationships among the different topological trees constructed using cp and nuclear genes suggest the existence of hybridization, incomplete lineage sorting, and/or interspecific introgression (Yang et al., 2013; Gatesy et al., 2019), and complex genetic relationships can be revealed by phylogenetic analyses based on cp and nuclear genes. However, few studies have focused on the infrageneric taxonomic system, species delimitation, and interspecies systematic relationships of *Leontopodium* based on cp and nuclear genes.

Therefore, in this study, the structure, gene content, and general characteristics of 43 cp genomes were compared and analyzed in detail to explore the evolution of the cp genome. Subsequently, phylogenetic trees were constructed using cp genomes and nrDNA to identify markers that are more effective for phylogenetic resolution, examine the phylogenetic relationships within *Leontopodium* from China, and further determine the phylogenetic position of *Leontopodium*. Finally, together with morphological characteristics, the infrageneric taxonomy system, species delimitation, and interspecies systematic relationships of *Leontopodium* were identified and discussed.

## 2 Materials and methods

### 2.1 Taxon sampling

The sequences used in this study included both new sequences and previously published sequences. Leaf materials of 43 individuals representing 30 taxa (one *Filago*, three *Gamochaeta*, and 26 *Leontopodium*) were obtained. Most materials used in this study were collected from natural populations in China, and voucher specimens were deposited in the herbarium of Zhengzhou University (ZZU; Zhengzhou, China). Leaf materials of a few taxa were obtained from herbarium specimens of PE (Institute of Botany, Chinese Academy of Sciences, Beijing, China). Detailed information on the samples is provided in **Table 1**. The NCBI database accession numbers of the new sequences (cp genomes, ITS, and ETS) in this study are shown in **Table 2**. Furthermore, the cp genome of *Gamochaeta coarctata* Kerguélen (MK570596) was acquired for phylogenetic reconstruction. Previously published nrDNA sequences (205 ITS and 205 ETS sequences of 101 taxa, including four *Antennaria*, 33 *Filago*, 30 Gamochaeta, and 34 *Leontopodium*) of the same individuals were obtained from the NCBI database and are listed in **Supplementary Table 1**. Based on phylogenetic results reported by Fu et al. (2016); Huang et al. (2016); Panero and Crozier (2016); Mandel et al. (2019) and Zhang C. et al. (2021), *Calendula arvensis* M.Bieb. was used as an outgroup (NCBI accession numbers: cp genome, ON641308; ITS, GU818507; and ETS, GU818129).

**Table 1:** Voucher specimens and location information.

Taxa	Voucher specimen	Location
*Filago arvensis* L. 1	K.Y. Lang et al. 127 (PE)	Aletai, Xinjiang, China
*F. arvensis* 2	E.E. Yayhino s.n. (PE)	Russia
*Gamochaeta norvegica* (Gunnerus) Y.S.Chen & R.J.Bayer	K.Y. Lang et al. 137 (PE)	Aletai, Xinjiang, China
*G. pensylvanica* (Willd.) Cabrera 1	Q.F. Zhao et al. ZSX1910094 (ZZU)	Chengdu, Sichuan, China
*G. pensylvanica* 2	Q.F. Zhao et al. zsx20191003 (ZZU)	Chengdu, Sichuan, China
*G. sylvatica* Fourr.	B. Deylova s.n. (PE)	Czech Republic
*Leontopodium andersonii* C.B.Clarke 1	Y. He et al. BNU2018YN396 (BNU, ZZU)	Luquan, Yunnan, China
*L. andersonii* 2	Q.F. Zhao et al. ZSX1907163 (ZZU)	Xianggelila, Yunnan, China
*L. artemisiifolium* Beauverd 1	X. Li 78613 (PE)	Jinchuan, Sichuan, China
*L. artemisiifolium* 2	Q.F. Zhao et al. ZSX1907131 (ZZU)	Yulong, Yunnan, China
*L. calocephalum* Beauverd 1	Q.F. Zhao et al. ZSX1907297 (ZZU)	Xianggelila, Yunnan, China
*L. calocephalum* 2	Q.F. Zhao et al. ZSX1907342 (ZZU)	Deqin, Yunnan, China
*L. calocephalum* 3	Q.F. Zhao et al. ZSX1908155 (ZZU)	Kangding, Sichuan, China
*L. calocephalum* 4	Q.F. Zhao et al. ZSX1908251 (ZZU)	Emei, Sichuan, China
*L. campestre* Hand.-Mazz.	A.R. Li & J.N. Zhu 5825 (PE)	Tulufan, Xinjiang, China
*L. conglobatum* Hand.-Mazz.	H.Z. Ma s.n. (PE)	Zhangjiakou, Hebei, China
*L. dedekensii* Beauverd 1	S.X. Zhu et al. DS15016 (ZZU)	Mianning, Sichuan, China
*L. delavayanum* Hand.-Mazz.	Q.F. Zhao et al. ZSX1907094 (ZZU)	Yulong, Yunnan, China
*L. fangingense* Y.Ling	Wuling Mountain Exped. 1322 (PE)	Tongren, Guizhou, China
*L. forrestianum* Hand.-Mazz. 1	K.M. Feng 7881 (PE)	Yunnan, China
*L. franchetii* Beauverd 1	Q.F. Zhao et al. ZSX1908189 (ZZU)	Yajiang, Sichuan, China
*L. franchetii* 2	Q.F. Zhao et al. ZSX1908190 (ZZU)	Yajiang, Sichuan, China
*L. giraldii* Diels	Y.S. Chen 8128 (PE)	Meixian, Shanxi, China
*L. himalayanum* DC. 1	Q.F. Zhao et al. ZSX1907334 (ZZU)	Deqin, Yunnan, China
*L. himalayanum* 2	Q.F. Zhao et al. ZSX1907337 (ZZU)	Deqin, Yunnan, China
*L. jacotianum* Beauverd 1	X.Y. Zhu et al. G10203 (ZZU)	Kangding, Sichuan, China
*L. japonicum* Miq. 1	S.X. Zhu et al., 20170801 (ZZU)	Jiyuan, Henan, China
*L. japonicum* var. *saxatile* Y.S.Chen 1	X.M. Xu et al. SC152 (ZZU)	Jinchuan, Sichuan, China
*L. japonicum* var. *saxatile* 2	X.M. Xu et al. SC153 (ZZU)	Jinchuan, Sichuan, China
*L. leontopodioides* Beauverd 1	R.C. Qin 5016 (PE)	Qinghe, Xinjiang, China
*L. longifolium* Y.Ling 1	Q.F. Zhao et al. ZSX1908154 (ZZU)	Kangding, Sichuan, China
*L. longifolium* 2	Q.F. Zhao et al. ZSX1908202 (ZZU)	Yajiang, Sichuan, China
*L. muscoides* Hand.-Mazz. 1	Q.F. Zhao et al. ZSX1908005 (ZZU)	Daocheng, Sichuan, China
*L. muscoides* 2	Q.F. Zhao et al. ZSX1908051 (ZZU)	Daocheng, Sichuan, China
*L. nanum* (Hook.f. & Thomson ex C.B.Clarke) Hand.-Mazz. 1	Y. Lu LY201801 (ZZU)	Xinjiang, China
*L. ochroleucum* Beauverd 1	Y. Lu LY201802 (ZZU)	Xinjiang, China
*L. ochroleucum* 2	K. Guo & D. Zheng 12372 (PE)	Xinjiang, China
*L. pusillum* (Beauverd) Hand.-Mazz. 1	X.M. Xu et al. BNU2019XZ052 (ZZU)	Gongbujiangda, Xizang, China
*L. sinense* Hemsl. ex F.B.Forbes & Hemsl. 1	Q.F. Zhao et al. ZSX1908001 (ZZU)	Daocheng, Sichuan, China
*L. smithianum* Hand.-Mazz.	X.M. Xu et al. XLM002 (ZZU)	Beijing, Beijing, China
*L. souliei* Beauverd 1	Q.F. Zhao et al. ZSX1908119 (ZZU)	Daocheng, Sichuan, China
*L. stracheyi* C.B.Clarke ex Hemsl. 1	S.X. Zhu et al. DS15006 (ZZU)	Lixian, Sichuan, China
*L. wilsonii* Beauverd 1	Q.F. Zhao et al. ZSX1908223 (ZZU)	Baoxing, Sichuan, China

**Table 2:** Accession numbers of new sequences (chloroplast genomes, ITS, and ETS) and the features of chloroplast genomes.

Taxa	Accession numbers			Genome size (bp)	LSC length (bp)	SSC length (bp)	IR length (bp)	Number of genes				G + C (%)			
	Cp	ITS	ETS					Total number of genes	CDS	tRNAs	rRNAs	Total genome	LSC	SSC	IR
*Filago arvensis 1*	OP963955	OP950237	OP946404	151,511	83,508	18,311	24,846	132	85	37	8	37.3%	35.2%	30.9%	43.1%
*F. arvensis 2*	OP963956	OP950238	OP946405	151,458	83,513	18,271	24,837	132	85	37	8	37.3%	35.2%	30.9%	43.0%
*Gamochaeta norvegica*	OP963957	OP950239	OP946406	151,348	83,986	18,300	24,531	132	85	37	8	37.3%	35.3%	31.0%	43.1%
*G. pensylvanica 1*	OP963958	OP950240	OP946407	151,574	83,632	18,244	24,849	132	85	37	8	37.3%	35.3%	31.0%	43.1%
*G. pensylvanica 2*	OP963959	OP950241	OP946408	151,573	83,632	18,243	24,849	132	85	37	8	37.3%	35.3%	31.0%	43.1%
*G. sylvatica*	OP963960	OP950242	OP946409	151,414	83,983	18,369	24,531	132	85	37	8	37.3%	35.3%	31.0%	43.1%
*Leontopodium andersonii 1*	OP963961	OP950243	OP946410	151,128	83,362	18,052	24,857	131	85	36	8	37.3%	35.3%	31.0%	43.1%
*L. andersonii 2*	OP963962	OP950244	OP946411	151,068	83,303	18,051	24,857	132	85	37	8	37.3%	35.3%	31.0%	43.1%
*L. artemisiifolium 1*	OP963963	OP950245	OP946412	151,073	83,281	18,068	24,862	131	85	36	8	37.3%	35.3%	31.0%	43.1%
*L. artemisiifolium 2*	OP963964	OP950246	OP946413	151,133	83,364	18,057	24,856	132	85	37	8	37.3%	35.2%	31.0%	43.1%
*L. calocephalum 1*	OP963965	OP950247	OP946414	151,100	83,327	18,061	24,856	132	85	37	8	37.3%	35.3%	31.0%	43.1%
*L. calocephalum 2*	OP963966	OP950248	OP946415	151,129	83,357	18,060	24,856	132	85	37	8	37.3%	35.2%	31.0%	43.1%
*L. calocephalum 3*	OP963967	OP950249	OP946416	151,102	83,330	18,060	24,856	132	85	37	8	37.3%	35.3%	31.0%	43.1%
*L. calocephalum 4*	OP963968	OP950250	OP946417	151,094	83,321	18,061	24,856	132	85	37	8	37.3%	35.3%	31.0%	43.1%
*L. campestre*	OP963969	OP950251	OP946418	151,122	83,368	18,042	24,856	132	85	37	8	37.3%	35.2%	31.0%	43.1%
*L. conglobatum*	OP963970	OP950252	OP946419	151,077	83,313	18,050	24,857	132	85	37	8	37.3%	35.3%	31.0%	43.1%
*L. dedekensii 1*	OP963971	OP950253	OP946420	151,071	83,305	18,052	24,857	132	85	37	8	37.3%	35.3%	31.0%	43.1%
*L. delavayanum*	OP963972	OP950254	OP946421	151,138	83,370	18,056	24,856	132	85	37	8	37.3%	35.2%	31.0%	43.1%
*L. fangingense*	OP963973	OP950255	OP946422	150,754	83,278	17,764	24,856	132	85	37	8	37.4%	35.3%	31.2%	43.1%
*L. forrestianum 1*	OP963974	OP950256	OP946423	151,143	83,369	18,092	24,841	132	85	37	8	37.3%	35.2%	30.09%	43.1%
*L. franchetii 1*	OP963975	OP950257	OP946424	151,095	83,338	18,057	24,850	132	85	37	8	37.3%	35.2%	31.0%	43.1%
*L. franchetii 2*	OP963976	OP950258	OP946425	151,157	83,387	18,058	24,856	132	85	37	8	37.3%	35.2%	31.0%	43.1%
*L. giraldii*	OP963977	OP950259	OP946426	151,093	83,321	18,060	24,856	132	85	37	8	37.3%	35.3%	31.0%	43.1%
*L. himalayanum 1*	OP963978	OP950260	OP946427	151,107	83,341	18,054	24,856	132	85	37	8	37.3%	35.3%	31.0%	43.1%
*L. himalayanum 2*	OP963979	OP950261	OP946428	151,128	83,365	18,051	24,856	132	85	37	8	37.3%	35.2%	31.0%	43.1%
*L. jacotianum 1*	OP963980	OP950262	OP946429	151,074	83,308	18,052	24,857	132	85	37	8	37.3%	35.3%	31.0%	43.1%
*L. japonicum 1*	OP963981	OP950263	OP946430	151,117	83,352	18,053	24,856	132	85	37	8	37.3%	35.3%	31.0%	43.1%
*L. japonicum* var. *saxatile 1*	OP963982	OP950264	OP946431	151,063	83,299	18,050	24,857	132	85	37	8	37.3%	35.3%	31.0%	43.1%
*L. japonicum* var. *saxatile 2*	OP963983	OP950265	OP946432	151,063	83,299	18,050	24,857	132	85	37	8	37.3%	35.3%	31.0%	43.1%
*L. leontopodioides 1*	OP963984	OP950266	OP946433	151,138	83,370	18,056	24,856	132	85	37	8	37.3%	35.2%	31.0%	43.1%
*L. longifolium 1*	OP963985	OP950267	OP946434	151,072	83,305	18,051	24,858	132	85	37	8	37.3%	35.3%	31.0%	43.1%
*L. longifolium 2*	OP963986	OP950268	OP946435	151,136	83,367	18,057	24,856	132	85	37	8	37.3%	35.2%	31.0%	43.1%
*L. muscoides 1*	OP963987	OP950269	OP946436	151,135	83,367	18,056	24,856	132	85	37	8	37.3%	35.2%	31.0%	43.1%
*L. muscoides 2*	OP963988	OP950270	OP946437	151,118	83,349	18,057	24,856	132	85	37	8	37.3%	35.3%	31.0%	43.1%
*L. nanum 1*	OP963989	OP950271	OP946438	151,132	83,362	18,058	24,856	132	85	37	8	37.3%	35.2%	31.0%	43.1%
*L. ochroleucum 1*	OP963990	OP950272	OP946439	151,141	83,372	18,057	24,856	132	85	37	8	37.3%	35.2%	31.0%	43.1%
*L. ochroleucum 2*	OP963991	OP950273	OP946440	151,097	83,337	18,060	24,850	132	85	37	8	37.3%	35.2%	31.0%	43.1%
*L. pusillum 1*	OP963992	OP950274	OP946441	151,130	83,364	18,054	24,856	132	85	37	8	37.3%	35.2%	31.0%	43.1%
*L. sinense 1*	OP963993	OP950275	OP946442	151,072	83,307	18,051	24,857	132	85	37	8	37.3%	35.3%	31.0%	43.1%
*L. smithianum*	OP963994	OP950276	OP946443	151,103	83,319	18,072	24,856	132	85	37	8	37.3%	35.3%	31.0%	43.1%
*L. souliei 1*	OP963995	OP950277	OP946444	151,138	83,367	18,059	24,856	132	85	37	8	37.3%	35.2%	31.0%	43.1%
*L. stracheyi 1*	OP963996	OP950278	OP946445	151,131	83,363	18,054	24,857	132	85	37	8	37.3%	35.2%	31.0%	43.1%
*L. wilsonii 1*	OP963997	OP950279	OP946446	151,105	83,330	18,063	24,856	132	85	37	8	37.3%	35.3%	31.0%	43.1%

### 2.2 DNA extraction, genome sequencing, and assembly

We used a modified cetyltrimethylammonium bromide (CTAB) method to extract high-quality DNA (Doyle and Doyle, 1987), which was then purified using the Wizard^®^ DNA cleanup system (Promega, Madison, WI, USA). DNA quality was assessed using a NanoDrop spectrophotometer (Thermo Scientific, Carlsbad, CA, USA), and DNA integrity was evaluated by electrophoresis on a 1% (w/v) agarose gel. A DNA library was prepared using the NEB Next Ultra DNA Library Prep Kit for Illumina (San Diego, CA, USA). Libraries for paired-end 150-bp sequencing were analyzed on an Illumina NovaSeq 6000 platform (Novogene Co., Ltd., Tianjin, China) to generate approximately 10 GB of data for each sample. Raw reads were filtered using SOAPnuke to remove sequencing adaptors and low-quality bases (Chen et al., 2018). The filtered reads were assembled using GetOrganelle (Jin et al., 2020) with a range of 21, 45, 65, 85, and 105 k-mers for plastomes and 35, 85, and 115 k-mers for nrDNA. Subsequently, ITS and ETS sequences were uploaded to the NCBI GenBank database (accession numbers are listed in **Table 2**).

### 2.3 Cp genome annotation and comparative analysis

The plastome sequences were initially annotated using Geneious Prime 2020.1.2 (https://www.geneious.com) by referring to the cp genome sequence of *Anaphalis sinica* Hance (KX148081), *Anaphalis margaritacea* var. *yedoensis* Ohwi (LC656264), and *G. coarctata* (MK570596). Annotations of protein-coding sequences were manually checked based on the open reading frame. Transfer RNA (tRNA) genes were verified using the online tRNAscan-SE tool with default settings (Lowe and Chan, 2016). All cp genome sequences have been deposited in the NCBI GenBank database (accession numbers are listed in **Table 2**). The complete cp genome was visualized using OGDRAW (Greiner et al., 2019). The mVISTA program in Shuffle-LAGAN mode was used to compare the cp genomes, using *Filago arvensis* L. (OP963955) as the reference (Frazer et al., 2004). The junctions and borders of the IR regions were visualized using IRScope (Amiryousefi et al., 2018). DnaSP version 6 was used to calculate the nucleotide variability (Pi) among the cp genomes (Rozas et al., 2017).

### 2.4 Phylogenetic analysis

Phylogenetic topology was constructed based on five matrices: complete cp sequences, coding genes of chloroplast genomes, ITS, ETS, and concatenated sequences of ITS and ETS. An online version of MAFFT (Katoh et al., 2019) was used to align the datasets. Phylogenetic analyses were performed using maximum likelihood (ML) and Bayesian inference (BI) methods using IQ-TREE v1.6.12 and MrBayes 3.2.2, respectively (Ronquist et al., 2012; Nguyen et al., 2015). The best-fitting model of nucleotide substitutions was determined using ModelFinder in PhyloSuite v1.2.2 (Zhang D. et al., 2020). ML analyses were performed using IQ-TREE with 1,000 bootstrap (BS) replicates. BI analysis was run for 5,000,000 generations and sampled every 5,000 generations; the first 25% of the trees were discarded as burn-in. Trees were selected based on a 50% majority-rule consensus to estimate posterior probabilities (PP). The effective sample size (>200) was determined using Tracer v1.7 (Rambaut et al., 2018). The reconstructed trees were visualized using Figtree V.1.4.2 (Rambaut, 2014) and TreeGraph 2 (Stöver and Müller, 2010).

## 3 Results

### 3.1 Characteristics of cp genomes

**Figure 1:**
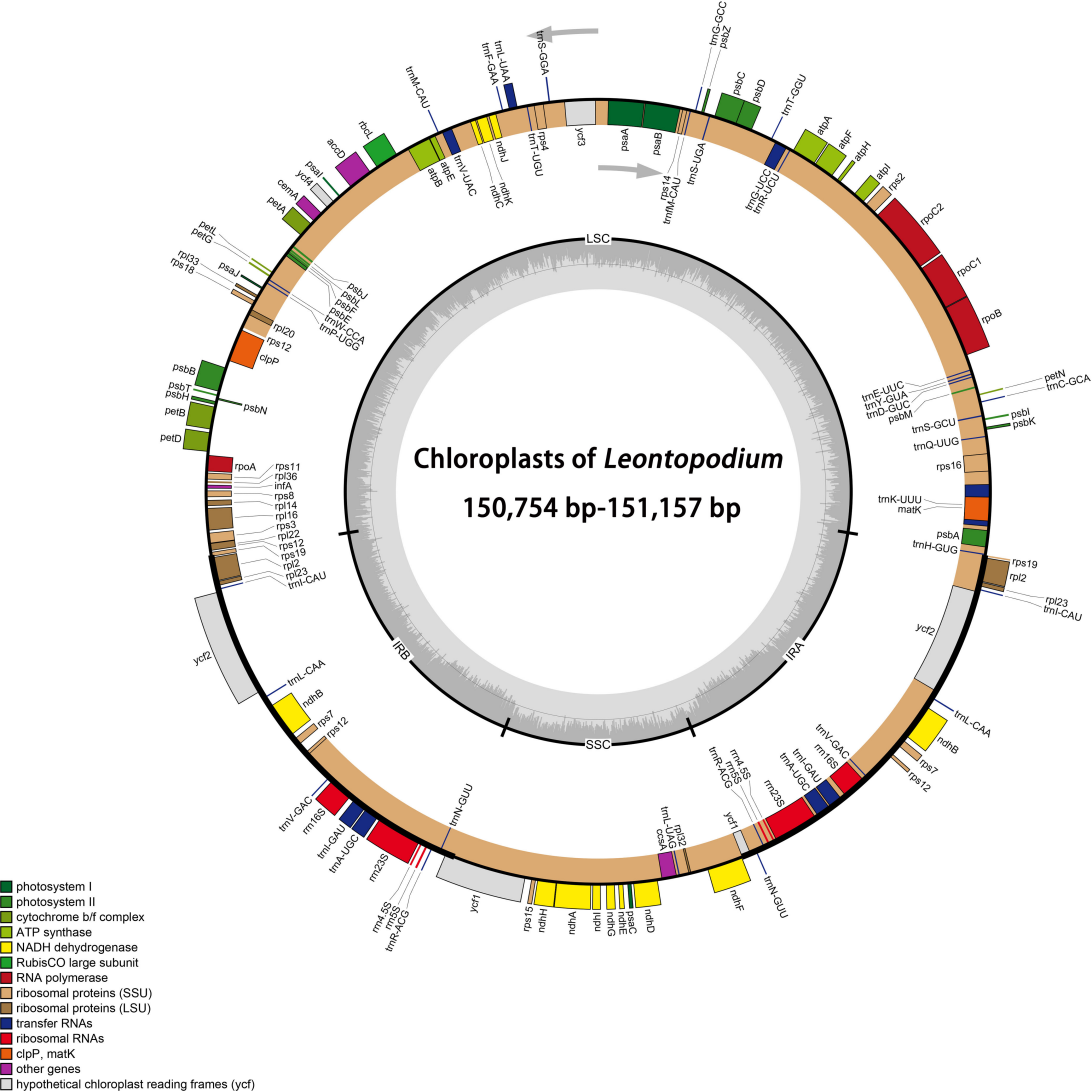
Chloroplast genome gene map of *Leontopodium*. Gray arrows indicate the direction of gene transcription. Genes belonging to different functional groups are marked with different colors. The dashed area in the inner circle indicates the GC content of the chloroplast genome; small single copy (SSC), large single copy (LSC), and inverted repeats (IRA and IRB) are indicated.

A total of 43 cp genomes (30 taxa, including one *Filago*, three *Gamochaeta*, and 26 *Leontopodium*) were compared. All cp genomes had a typical quadripartite structure: an LSC, SSC, and two IRs (**Figure 1**). Among the 43 samples, the total length of cp genomes ranged from 150,754 bp (*L. fangingense*, OP963973) to 151,574 bp (*G. pensylvanica*, OP963958) (**Table 2**). The lengths of LSC, SSC, and IRs ranged from 83,278 bp (*L. fangingense*) to 83,986 bp (*G. norvegica*, OP963957); 17,764 bp (*L. fangingense*) to 18,369 bp (*G. sylvatica*, OP963960); and 24,531 bp (*G. norvegica*) to 24,862 bp (*L. artemisiifolium*, OP963963), respectively (**Table 2**). The lengths of the complete cp genomes, LSC, SSC, and IRs of *Leontopodium* ranged from 150,754 bp (*L. fangingense*) to 151,157 bp (*L. franchetii*, OP963976), 83,278 bp (*L. fangingense*) to 83,387 bp (*L. franchetii*), 17,764 bp (*L. fangingense*) to 18,092 bp (*L. forrestianum*, OP963974), and 24,850 bp (*L. franchetii*) to 24,862 bp (*L. artemisiifolium*), respectively (**Table 2**). Compared with *Filago* and *Gamochaeta*, *Leontopodium* had longer whole cp genomes and LSC and SSC regions; however, the length of the IRs was slightly shorter. In *Leontopodium*, the length of the SSC region varied more significantly (328 bp) than that of the LSC (109 bp) and IR (21 bp) regions. The cp genomes comprised 131–132 genes, including 85 protein-coding, eight rRNA, and 36–37 tRNA genes (**Table 2**), two of which, *L. artemisiifolium* 1 and *L. andersonii* 1, lacked the *trnT-GGU* gene. The total GC content of the cp genomes was highly similar (37.3%–37.4%). The GC content of IRs (43.0%–43.1%) was higher than that of the LSC (35.2%–35.3%) and SSC (30.09%–31.2%) regions (**Table 2**). Compared with other species of *Leontopodium*, the GC content of the total cp genome and SSC region in *L. fangingense* was higher.

### 3.2 Boundaries between IR and SC regions

**Figure 2:**
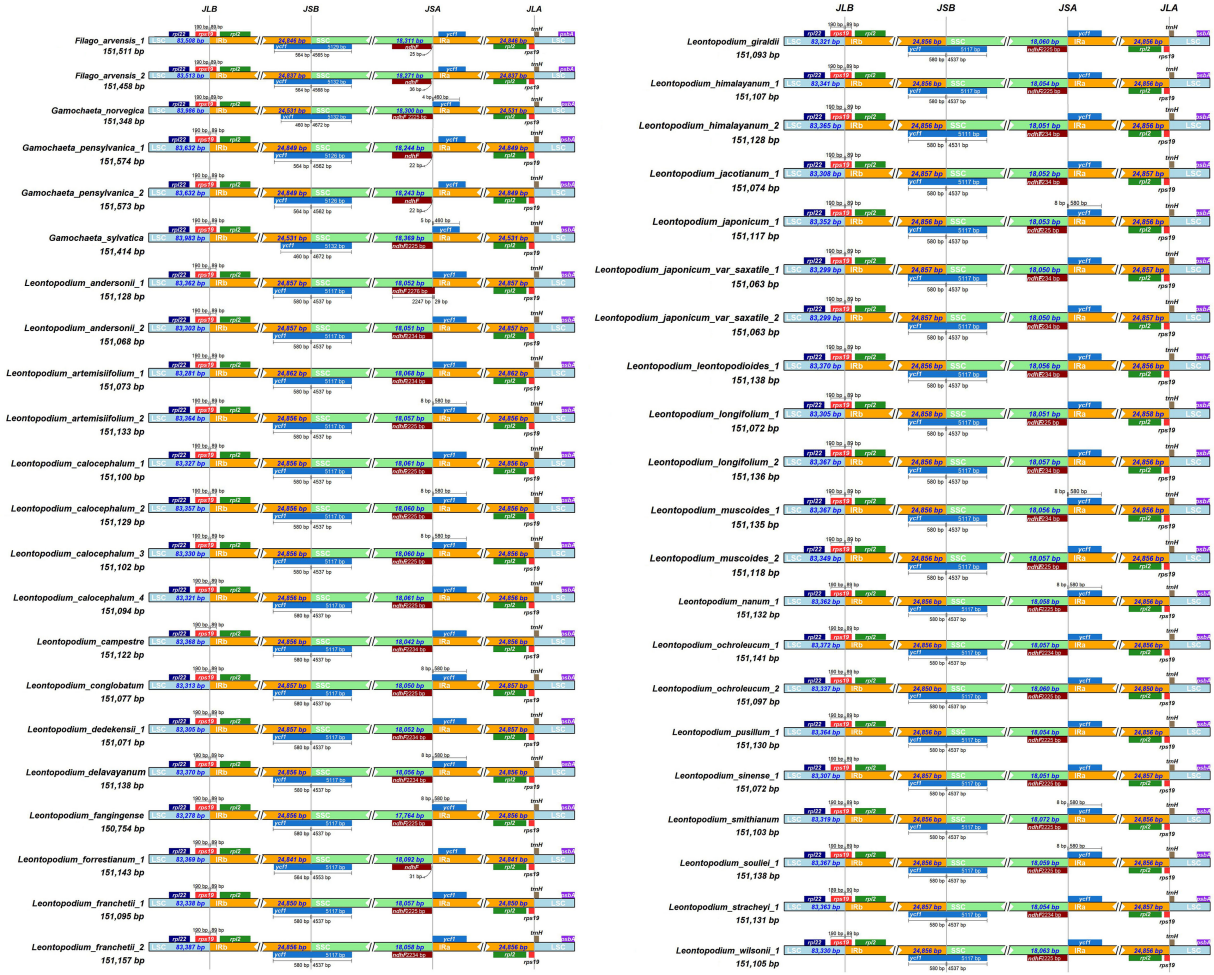
Comparison of the LSC, IR, and SSC border regions among the chloroplast genomes of *Leontopodium*, *Filago*, and *Gamochaeta*.

All 43 cp genomes were analyzed, and the differences among the junctions of the LSC/IRb (JLB), IRb/SSC (JSB), SSC/IRa (JSA), and IRa/LSC (JLA) regions were compared (**Figure 2**). Most cp genomes have similar characteristics. The junctions of the LSC/IRb regions in all the 43 cp genomes were located at *rps19*. Other than *L. stracheyi*, all taxa had 190 bp of *rps19* in the LSC region and 89 bp in the IRb region. *ycf1* was located at the IRb/SSC junction in all samples. *F. arvensis* had 564 bp *ycf1* in the IRb region and 4,565–4,568 bp in the LSC region. The taxa of *Gamochaeta* had 460–564 bp *ycf1* in the IRb region and 4,562–4,672 bp in the LSC region. In *Leontopodium*, except for *L. forrestianum* and *L. himalayanum* 2, all samples had 580 bp of *ycf1* in the IRb regions and 4537 bp in the LSC regions. *L. forrestianum* had 564 bp of *ycf1* in the IRb region and 4,553 bp in the LSC region. *L. himalayanum* 2 had 580 bp of *ycf1* in the IRb region and 4,531 bp in the LSC region. The *ndhF* and ψ*ycf1* genes were detected at the SSC/IRa boundary. Except for *L. andersonii* 1, the *ndhF* of all samples was located entirely in the SSC region. *L. andersonii* 1 had 29 bp of *ndhF* in the IRa region. The ψ*ycf1* gene of some samples, such as *G. norvegica*, *G. sylvatica*, *L. artemisiifolium* 2, *L. conglobatum*, *L. delavayanum*, and *L. japonicum*, crossed the boundary of the SSC and IRa regions, with 4, 5, or 8 bp extending into the SSC region. The ψ*rps19* and *trnH* genes were detected in the IRa/LSC junctions of all 43 cp genomes.

### 3.3 Comparative genomic analysis and divergence hotspot regions

**Figure 3:**
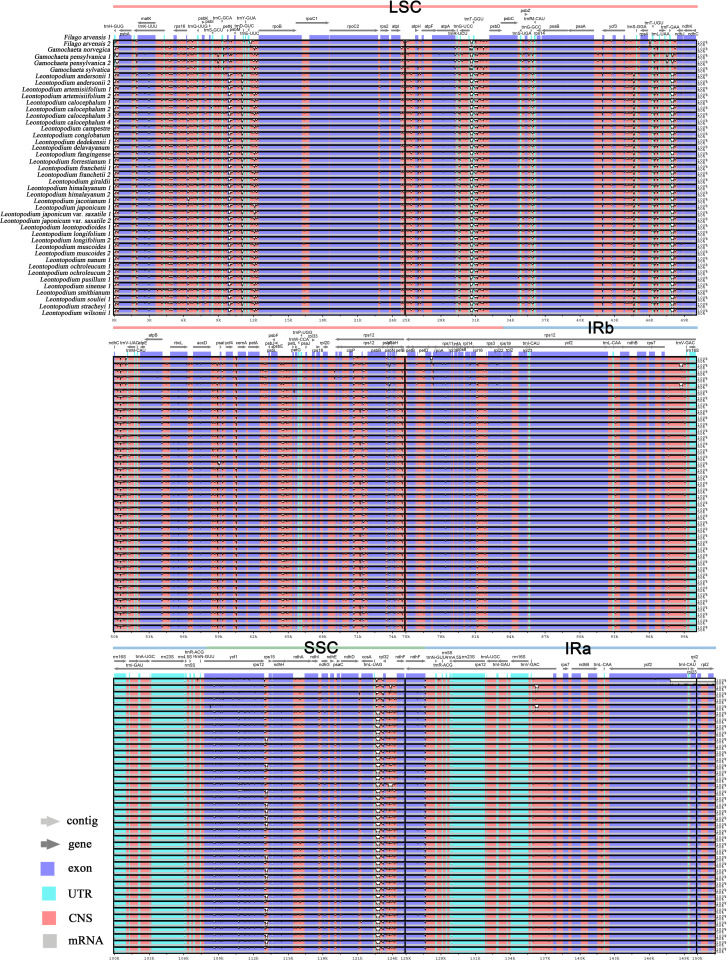
Sequence alignment of 43 chloroplast genomes using the mVISTA program, with *F. arvensis* as a reference. Genome regions are color-coded as protein-coding, rRNA-coding, tRNA-coding, or conserved noncoding sequences. The vertical scale indicates percentage identity, ranging from 50% to 100%. Regions with sequence variations among the chloroplast genomes are denoted in white.

**Figure 4:**
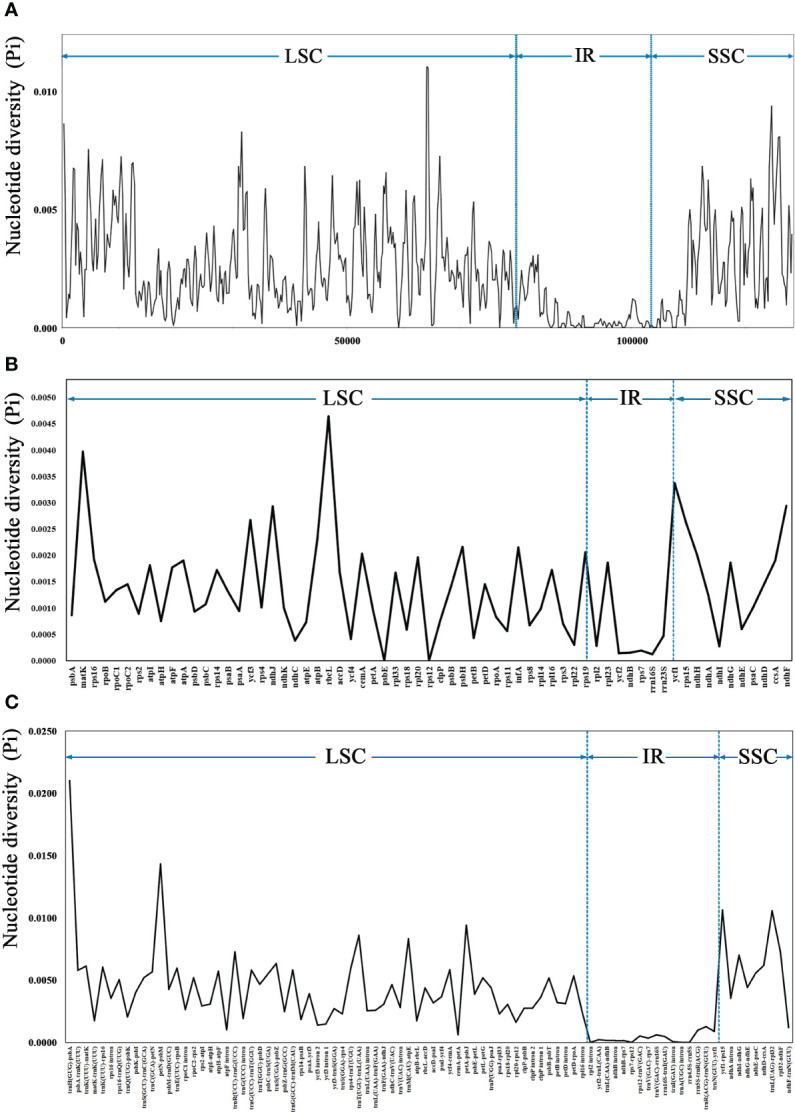
Nucleotide diversity (Pi) of 43 complete chloroplast genomes. **(A)** Sliding window analysis using a window length of 500 bp and step size of 200 bp. **(B)** Coding regions (aligned length >200 bp). **(C)** Noncoding regions (aligned length of >200 bp). The vertical dotted lines divide the approximate boundaries of LSC, IR, and SSC.

The sequence divergence of the 43 cp genomes was comprehensively analyzed using the mVISTA program, with *F. arvensis* as a reference. Overall, the 43 cp genomes exhibited relatively high diversity (**Figure 3**), with genic regions being more conserved than intergenic spacer (IGS) regions. Most genic regions were highly convergent; the only divergent regions were detected in *matK*, *atpA*, *rbcL*, *accD*, *rpoA*, *ycf1*, *ndhH*, *ndhG*, and *ndhF*. The highest divergence was observed in IGS regions such as *trnH (GUG)-psbA*, *trnK (UUU)-rps16*, *rps16-trnQ (UUG)*, *petN-psbM*, *trnE (UUC)-rpoB*, and *atpI-atpH*. The LSC and SSC regions showed a higher level of sequence divergence than the IR region. Moreover, the cp genome structure of *Leontopodium* exhibited higher consistency and was obviously different from that of *Filago* and *Gamochaeta* in some regions, such as *matk*, *trnK (UUU)-rps16*, *rps16-trnQ (UUG)*, *trnC (GCA)-petN*, *petN-psbM*, and *trnE (UUC)-rpoB*. In *Leontopodium*, divergent regions were detected in the *rps16-trnQ (UUG)*, *trnE (UUC)-rpoB*, *ycf1-rps15*, and *clpP* genes. Sliding window analyses of 43 cp genomes indicated that most of the variation occurred in the LSC and SSC regions, which exhibited high nucleotide variability (**Figure 4A**). In addition, 65 coding regions (aligned length >200 bp) and 88 non-coding regions (aligned length >200 bp) were extracted, and nucleotide variability was calculated (**Supplementary Tables 2**, **3**). Moreover, the nucleotide diversity (Pi) values of the 65 coding regions and 88 non-coding regions were compared across the 43 cp genomes (**Figures 4B, C**). In the coding regions, the loci with the largest variation were *ndhH*, *cemA*, *rps19*, *infA*, *psbH*, *atpB*, *rps15*, *ycf3*, *ndhJ*, *ndhF*, *ycf1*, *matK*, and *rbcL* (Pi >0.002; **Figure 4B**); whereas in non-coding regions, the loci with the largest variation were *trnK (UUU)-rps16*, *trnK (UUU)-matK*, *ndhD-ccsA*, *trnS (UGA)-psbZ*, *ndhI-ndhG*, *trnR (UCU)-trnG (UCC)*, *rpl32-ndhF*, *trnM (CAU)-atpE*, *trnT (UGU)-trnL (UAA)*, *petA-psbJ*, *trnL (UAG)-rpl32*, *ycf1-rps15*, *petN-psbM*, and *trnH (GUG)-psbA* (Pi >0.006; **Figure 4C**).

### 3.4 Phylogenetic results of cp genomes

**Figure 5:**
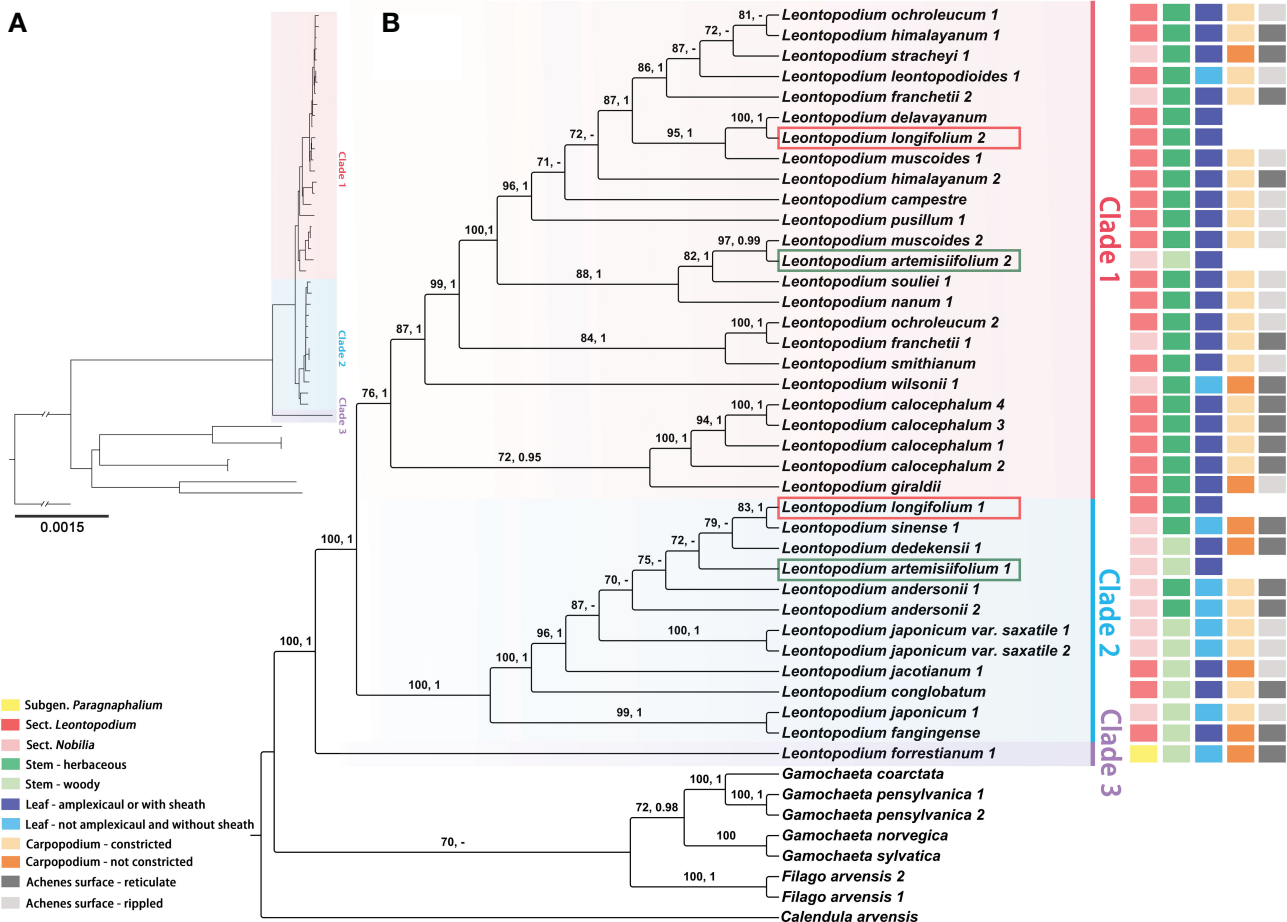
Phylogenetic trees of *Leontopodium*, *Filago*, and *Gamochaeta*, together with *Calendula arvensis* as an outgroup, were inferred from complete chloroplast genomes. **(A)** Topology of the ML tree. **(B)** ML tree with bootstrap values of ML and posterior probabilities of BI shown at each node. Bootstrap values higher than 70 and posterior probabilities higher than 0.90 are indicated on branches. “–” means that the bootstrap value/posterior probability is less than 70/0.90. The taxonomy system of *Leontopodium* in Lin (1965) and the morphological characteristics of stems, leaves, and achenes are mapped on the right side.

The sequence characteristics and nucleotide substitution models for ML and BI phylogenetic analyses of different datasets (complete cp genomes, coding genes of chloroplast genomes, ITS, ETS, and concatenated sequences of ITS and ETS) are presented in **Supplementary Table 4**. In the phylogenetic trees that were inferred using complete cp genomes (**Figure 5** and **Supplementary Figure 1**), *Filago* and *Gamochaeta* were sister groups and formed one clade, respectively, with strong support [*Filago*, ML bootstrap value (BS) = 100, Bayesian posterior probabilities (PP) = 1; *Gamochaeta*, BS = 72, PP = 0.98]. *Leontopodium* species were clustered into one clade (BS = 100, PP = 1) as a sister clade to the species to *Filago* and *Gamochaeta* species. In addition, three main clades were formed by the *Leontopodium* species (**Figure 5** and **Supplementary Figure 1**). Clade 3 was a sister clade to Clade 1 and Clade 2, and included only one species, *L. forrestianum*. Clade 1 and Clade 2 included 17 and 10 taxa, respectively. However, in the phylogenetic trees constructed using complete cp genomes (**Figure 5** and **Supplementary Figure 1**), the individuals of two species, *L. artemisiifolium* and *L. longifolium*, were in different main clades (Clade 1 and Clade 2). In Clade 1, the individuals of *L. franchetii*, *L. himalayanum*, *L. muscoides*, and *L. ochroleucum*, were not clustered together. Similar results were obtained for *L. andersonii* and *L. japonicum* in Clade 2. The topological structures of the phylogenetic trees inferred using coding genes of chloroplast genomes (**Supplementary Figure 2**) were different from those of complete cp genome trees. *F. arvensis*, *G. pensylvanica*, and *G. coarctata* formed one calde, which was sister to *G. norvegica* and *G. pensylvanica*. *Leontopodium* species were divided into five main clades; *L. forrestianum*, *L. giraldii*, and *L. calocephalum* formed independent clades, respectively (**Supplementary Figure 2**). The species in Clades II and V (coding gene tree, **Supplementary Figure 2**) were identical to those in Clades 2 and 3 (complete genome tree, **Figure 5**), respectively. The species in Clade 1 (**Figure 5**) were identical to those in Clades 1, 3, and 4 (**Supplementary Figure 2**). In addition, the support values of coding genes trees were lower.

### 3.5 Phylogenetic results of nuclear genes

**Figure 6:**
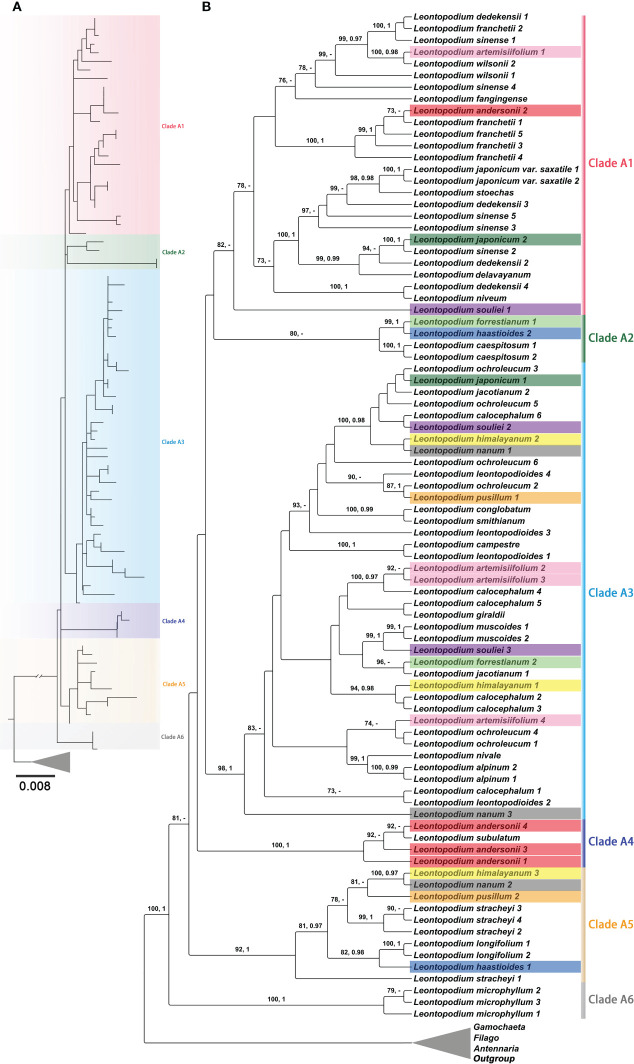
Partial phylogenetic trees of *Leontopodium* and its closely related genera, together with *Calendula arvensis* as an outgroup, were inferred from concatenated ITS and ETS sequences. **(A)** Topology of the ML tree. **(B)** ML tree, with bootstrap values of ML and posterior probabilities of BI shown at each node. Bootstrap values higher than 70 and posterior probabilities higher than 0.90 are indicated on branches. “–” means that the bootstrap value/posterior probability is less than 70/0.90.

In the phylogenetic trees inferred using ITS, ETS, and the concatenated sequences of ITS and ETS (**Figure 6** and **Supplementary Figures 3**-**5**), *Leontopodium* species were clustered into one clade, with strong support (BS = 100; PP = 1). *Leontopodium* was sister to *G. norvegica* and *G. sylvatica* in the phylogenetic trees constructed using ITS and concatenated sequences of ITS and ETS (**Figure 6** and **Supplementary Figures 3**, **5**). However, in the ETS phylogenetic tree, *Leontopodium* was sister to *Antennaria* taxa (**Supplementary Figure 4**). *Filago* species were also clustered into one clade, with strong support, in the phylogenetic trees inferred using nuclear genes; however, the taxa of *Antennaria* and *Gamochaeta* were divided into several main clades. For example, in the phylogenetic tree inferred from the concatenated sequences of ITS and ETS (**Supplementary Figure 5**), *Antennaria* species were in two main clades (Clades D1 and D2) and *Gamochaeta* species were in six main clades (Clades B1–B6). Furthermore, six main clades (Clades A1–A6) were formed by the *Leontopodium* species (**Figure 6** and **Supplementary Figure 5**). Clade A6 was a sister clade to the other clades and included only one species, *L. microphyllum*. Clade A2, Clade A4, and Clade A5 included 3, 2, and 6 taxa of *Leontopodium*, respectively. Most *Leontopodium* taxa were clustered in Clades A1 and A3. The samples of nine taxa, *L. artemisiifolium*, *L. andersonii*, *L. forrestianum*, *L. haastioides* Hand.-Mazz., *L. himalayanum*, *L. japonicum*, *L. nanum*, *L. pusillum* and *L. souliei*, were not clustered together.

## 4 Discussion

### 4.1 Characteristics of cp genomes and genetic variations

In this study, we evaluated 43 complete cp genomes of 30 taxa (one *Filago*, three *Gamochaeta*, and 26 *Leontopodium*), which were assembled *de novo* for the first time. The plastid genomes of the taxa analyzed here exhibited a typical quadripartite structure, with LSC and SSC regions separated by two IRs, and they were in the genome size range of land plants (**Figure 1** and **Table 2**) (Zheng et al., 2017). In addition, the structure, length, gene number, gene order, and GC content of the plastomes included in our analyses were consistent with those of previously reported Asteraceae cp genomes (Lee et al., 2016; Salih et al., 2017; Ou et al., 2019; Hatmaker et al., 2020; Kim et al., 2020; Loeuille et al., 2021; Pascual-Díaz et al., 2021; Thode et al., 2021; Peng et al., 2022; Yu et al., 2022), which may underscore the overall high stability of cp features at the lower taxonomic level. The cp genomes of *Filago*, *Gamochaeta*, and *Leontopodium* were well conserved in terms of gene number, gene order, and GC content (**Table 2**). The most remarkable differences among the three genera were the length of the complete cp genome, LSC, SSC, and IR (**Table 2**). Compared with *Filago* and *Gamochaeta*, *Leontopodium* had a shorter complete cp genome (150,754–151,157 bp), LSC (83,278–83,387 bp), and SSC (17,764–18,092 bp); in contrast, the IR region of *Leontopodium* was longer than that of *Filago* and *Gamochaeta* (**Table 2**). Furthermore, currently, the NCBI GenBank database contains about 30 cp genomes of Gnaphalieae, and the length of all complete cp genomes of Gnaphalieae reported was longer than 152 kb, such as *A. sinica* (152,718 bp, KX148081), *A. margaritacea* var. *yedoensis* (153,231 bp, LC656264), *Helichrysum italicum* (Roth) G.Don (152,431 bp, MK089797), *Pseudognaphalium oligandrum* (DC.) Hilliard & B.L.Burtt (152,797 bp, MK570591), and *P. sandwicensium* (Gaudich.) Anderb. (152,995 bp, MK570594). Pseudogenization of *trnT-GGU* was previously reported in *Cryptomeria japonica* D. Don., and *Pelargonium* × *hortorum* (Chumley et al., 2006; Hirao et al., 2008). Lee et al. (2017) determined that the *trnT-GGU* genes within the cp genomes of *A. sinica* and *L. leiolepis* were either pseudogenized or lost and suggested that mutations in the *trnT-GGU* gene might be used as indicators of generic and/or tribal relationships. In this study, *L. artemisiifolium* 1 and *L. andersonii* 1 lacked the *trnT-GGU* gene (other samples of these two species had the *trnT-GGU* gene), indicating that the loss of *trnT-GGU* genes was not associated with species circumscription in *Leontopodium*. Moreover, Abdullah et al. (2021) performed a broad analysis of the *trnT−GGU* gene of Asteraceae and found this gene to be a pseudogene in the Asteraceae core, which was linked to an insertion event within the 5′acceptor stem and was not associated with ecological factors such as habit, habitat, and geographical distribution of the species.

In general, the expansion and contraction of IR regions are related to variation in genome length (Raubeson et al., 2007; Wang and Messing, 2011), which is also considered a type of phylogenetic information (Menezes et al., 2018). In the present study, 43 cp genomes were analyzed and the differences between the boundary regions of SC and IR were compared **(** **Figure 2**). The genes located at the junctions were well conserved among the 43 cp genomes: *rps19* in LSC/IRb, *ycf1* in IRb/SSC, *ndhF* and ψ*ycf1* in SSC/IRa, and ψ*rps19* and *trnH* in IRa/LSC. Except for IRb/SSC, the boundaries of LSC/IRb, SSC/Ira, and IRa/LSC were relatively stable. The boundaries of IRb/SSC of *Leontopodium* were different from those of *Filago* and *Gamochaeta*, but were relatively stable at the genus level (**Figure 2**). Owing to the instability of IRb/SSC, the cp genome experienced IR/SSC contraction and expansion, which may be related to the variation in genome length observed among the three genera. With shifts in the IR/SSC boundaries, IR regions in *Leontopodium* became longer than those of *Filago* and *Gamochaeta*, which suggests that the IR expansion occurred after the splitting of *Leontopodium* from its sister clade. IRs are thought to stabilize the plastome through homologous recombination-induced repair mechanisms (Wicke et al., 2011). The longer IRs of plastomes are hypothesized to contribute to plastome stabilization because their absence often coincides with severe changes in the gene order (Palmer and Thompson, 1982). However, the cp gene number, length, and order of *Leontopodium*, *Filago*, and *Gamochaeta* were conserved, which indicated that the difference in IR observed among the three genera was insignificant.

Despite the relatively stable and conserved length, structure, gene number, and gene order of the cp genomes, mutation hotspots have been detected. In this study, we used mVISTA to compare the cp genomes of *Filago*, *Gamochaeta*, and *Leontopodium* (**Figure 3**), and used DnaSP to analyze the percentage of variable loci in the whole cp genomes, 65 coding regions, and 88 non-coding regions (**Figure 4** and **Supplementary Tables S2**, **3**). The mVISTA program revealed a relatively high diversity of 43 cp genomes, with genic regions being more conserved than intergenic regions, which is typical of angiosperm cp genomes (Huo et al., 2019; Song et al., 2019; Zhang X. F. et al., 2021). Moreover, as observed in other taxa of Asteraceae (Kim et al., 2020; Loeuille et al., 2021; Pascual-Díaz et al., 2021; Thode et al., 2021; Peng et al., 2022; Yu et al., 2022), the variation in the IR regions was smaller than that observed in the SC regions in the 43 cp genomes analyzed in our study (**Figures 3**, **4**). In addition, the cp genome structure of *Leontopodium* showed high consistency and was obviously different from, that of *Filago* and *Gamochaeta* in some regions, such as *matk*, *trnK (UUU)-rps16*, *rps16-trnQ (UUG)*, *trnC (GCA)-petN*, *petN-psbM*, and *trnE (UUC)-rpoB* (**Figure 3**), which may reflect the phylogenetic relationships among species or genera. In terms of nucleotide diversity, hotspot mutation regions were non-coding, consistent with other cp genomes (Liu et al., 2019; Park et al., 2020; Smidt et al., 2020; Pascual-Díaz et al., 2021). A previous study that investigated *Leontopodium* phylogeny using cp gene markers, such as *matK*, *trnL*, and *trnL-trnF*, failed to resolve the phylogenetic relationships within the genus (Blöch et al., 2010). Our analyses revealed relatively low nucleotide diversities of *matK* (Pi = 0.00397), *trnL* (Pi = 0), and *trnL-trnF* (Pi = 0.00258) compared to other loci (**Figure 4** and **Supplementary Tables 2**, **3**), which explains the low support in the phylogenetic trees that were inferred using these genes (Blöch et al., 2010). Moreover, we detected 14 hotspots (Pi >0.006) in the non-coding regions, which could be used as candidate DNA barcodes for future studies (**Supplementary Table 3**). Hotspot regions in plants indicate evolution and can be used to distinguish between species or genera (Liu et al., 2019). Therefore, these variable regions may also be useful for assessing the phylogenetic relationships and interspecific differences between *Leontopodium* species.

### 4.2 The conflicts of gene trees

The extensive heterogeneity in nucleotide substitution rates among different plastid genes and gene groups is likely to contribute to the phylogenetic ambiguity (Zhang X. et al., 2020; Li et al., 2021). In our phylogenetic analyses, sequence variations differed between the complete cp genome and coding genes datasets (**Supplementary Table 4**), which led to the different topological structures. This suggests that attention should be paid to the effects of such heterogeneity when functional genes or plastid fragments are used to study the phylogenetic evolution of the *Leontopodium* cp genome. Guo et al. (2023) indicated that one of the biggest challenges in the field of phylogenomics is the selection of appropriate genomic data for species tree reconstruction. Plastome sequences generally possess slow rates of evolution, in some cases resulting in insufficient numbers of informative sites for resolving rapid radiation (Gitzendanner et al., 2018). The coding genes of chloroplast genomes have fewer informative sites than those of complete chloroplast genomes. Therefore, in our study, the phylogenetic trees inferred using coding genes of chloroplast genomes were more confusing and had lower support values compared with the trees inferred using complete chloroplast genomes, because the non-coding regions of chloroplast genomes could provide more informative sites phylogenetic analysis.

Additionally, topological trees constructed based on complete cp genomes and nrDNA were incongruent (**Figures 5**, **6** and **Supplementary Figures 1**, **3**-**5**). Because cp genes and nuclear genes belong to different genetic systems, topological conflicts among different gene trees are a common phenomenon in molecular phylogenetic studies (Zou and Ge, 2008). Frequent hybridization, introgression, horizontal gene transfer, polyploidy accompanied by apomixis, or rapid radiation may have contributed to evolutionary complexity (Zou and Ge, 2008; Liu et al., 2020), and complex phylogenetic relationships are commonly found in Asteraceae (Bayer et al., 2002; Núria et al., 2002; Hidalgo et al., 2006; Kim et al., 2007; Barker et al., 2009; Galbany-Casals et al., 2010; Zhao et al., 2010; Jara-Arancio et al., 2017). The phylogenetic relationships inferred using cp genomes and nrDNA within *Leontopodium* were complex and incongruent. *Leontopodium* species were divided into three main clades in the phylogenetic tree obtained from the cp genomes (**Figure 5**), whereas six main clades were obtained in the phylogenetic tree inferred using nuclear genes (**Figure 6**). Moreover, two species were clustered into different main clades in the cp tree, whereas nine taxa were clustered in the nrDNA tree (**Figures 5**, **6**). For example, the samples of *L. longifolium* were divided into different main clades in the cp tree (**Figure 5**) but were clustered together in the nrDNA tree (Clade A5) (**Figure 6**). In the phylogenetic tree inferred using nrDNA, the samples of *L. longifolium* had a close relationship with *L. himalayanum*, *L. nanum*, *L. pusillum*, *L. stracheyi*, and *L. haastioides* (**Figure 6**). In the cp tree, one sample of *L. longifolium* (*L. longifolium* 2) was included in Clade 1, together with *L. himalayanum*, *L. nanum*, *L. pusillum*, and *L. stracheyi* (the cp genome of *L. haastioides* was not included in our study), whereas the other sample of *L. longifolium* (*L. longifolium* 1) was closely related to *L. sinense* and L. *dedekensii* (**Figure 5**). Subsequently, we noticed that three samples (*L. longifolium* 1, *L. sinense* 1, and *L*. *dedekensii* 1) were all from southern Sichuan, China. This raises the possibility that hybridization occurred between *L. longifolium* and other species of *Leontopodium* in Clade 2 (**Figure 5**), most likely *L. sinense* or a closely related sympatric species. This caused the capture of a foreign chloroplast haplotype in *L. longifolium* and fixation of the *L. longifolium* ITS type. Another example of incongruence resulting from possible hybridization is *L. artemisiifolium*. The Asteraceae family is highly evolved and is in a stage of rapid differentiation, resulting in a large number of complex and polymorphic transitional taxa in the family, which leads to great difficulties in classification and phylogeny research (Bremer, 1994). The natural hybrids of *Leontopodium* in China remain controversial (Lin, 1965; Chen et al., 2011), which also indicates complex phylogenetic relationships within this genus. Furthermore, incomplete lineage sorting is a stochastic process that potentially occurs in groups evolving through rapid adaptive radiation. Thus, a phylogeny based on maternally inherited chloroplast genes may not always correspond to a nuclear-gene-based phylogeny (Takahashi et al., 2001; Fior et al., 2013).

### 4.3 Phylogenetic relationships between *Leontopodium* and its related genera

All phylogenetic trees inferred using cp genomes and nuclear genes indicated that *Leontopodium* is monophyletic. In the cp genome phylogenetic tree, *Filago* and *Gamochaeta* clustered together and were sister to *Leontopodium* (**Figure 5** and **Supplementary Figure 1**). In the phylogenetic trees constructed using nrDNA, *Filago* was monophyletic, but *Gamochaeta* was nested within *Antennaria* (**Supplementary Figures 3**-**5**). However, Nie et al. (2016) indicated that *Antennaria*, *Leontopodium*, and *Gamochaeta* were monophyletic, whereas *Filago* was nested with *Hesperevax* A.Gray, *Micropus* L., *Psilocarphus* Nutt., *Bombycilaena* (DC.) Smoljan., and *Evacidium* Pomel. The samples included in the phylogenetic analyses can influence the conclusions; for example, *A. chilensis* J.Rémy and *A. linearifolia* Wedd. were not included in the study by Nie et al. (2016), but our research included two taxa, which led to the controversy regarding the monophyly of *Antennaria*. In the ETS tree, *Leontopodium* was closely related to *A. chilensis*, *A. microphylla* Rydb., and *A. dioica* (L.) Gaertn. (**Supplementary Figure 4**). Nevertheless, in the phylogenetic trees constructed using ITS and concatenated sequences of nrDNA, *Leontopodium* is sister to *G. norvegica* and *G. sylvatica* (**Supplementary Figures 3**, **5**). Galbany-Casals et al. (2010) indicated that *Leontopodium* was sister to other genera of the FLAG clade; however, only one species of *Leontopodium* was analyzed in their study. Three species of *Leontopodium* and *Castroviejoa montelinasana* (Em.Schmid) Galbany were clustered into one clade, albeit with low support (Fu et al., 2016). Nie et al. (2016) indicated that *Leontopodium* was sister to *Chionolaena* DC., *Facelis* Cass., *Lucilia* Cass., *Micropsis* DC., *Stuckertiella* Beauverd, *Gamochaeta*, etc. Thus, the monophyly of *Leontopodium* is definite, but the relationships between this genus and closely related genera remain unclear. Moreover, more molecular data and taxa should be used to investigate this in the future.

### 4.4 Phylogenetic relationships within the genus *Leontopodium*

Compared with nrDNA trees, the support values of cp trees were higher and could also provide clearer infrageneric and interspecies systematic relationships for *Leontopodium* (**Figures 5**, **6** and **Supplementary Figures 1**-**5**). The relationships within the genus *Leontopodium* have been previously inferred based on morphological characteristics, such as the morphology and color of the pappus, the shape of bracteal leaves, the indumentum of leaves or achenes, and the types of stems (Handel-Mazzetti, 1928; Lin, 1965). However, our molecular phylogenetic results differed greatly from the morphological classifications. Thus, based on a literature review, natural populations, and specimens of *Leontopodium*, in the present study, the classifications and phylogenetic relationships of 26 *Leontopodium* taxa in China were discussed based on the cp phylogenetic tree and the main characteristics of the leaves, stems, and achenes (**Figure 5**). Both Handel-Mazzetti (1928) and Lin (1965) reported that two subgenera should be included in *Leontopodium* according to the morphology and color of the pappus, and our results of cp phylogenetic trees also indicated that subgen. *Paragnaphalium* was sister to subgen. *Leontopodium* (sect. *Nobilia* and sect. *Leontopodium*) (**Figure 5**). However, in the phylogenetic trees inferred using nrDNA (**Figure 6** and **Supplementary Figures 3**-**5**), *L. forrestianum* (subgen. *Paragnaphalium*) was nested within the subgen. *Leontopodium*. Therefore, at the subgeneric level, the results of the cp genome phylogenetic analysis were consistent with the classification results obtained from the morphological features of *Leontopodium*. Lin (1965) indicated the morphology of plant, indumentum, and pappus of *L. forrestianum* was similar to that of *Gnaphalium*, but the characters of capitulum and bracteal leave supported this species should be included in *Leontopodium*, which showed that this species was distantly related to other *Leontopodium* species. *L. forrestianum* was the basal taxon of *Leontopodium* in the phylogenetic tree inferred using the cp genomes (**Figure 5**). This phenomenon indicated that plastid genomes could better resolve basal phylogenetic relationships because of their conservative property, and particular morphological variation was consistent with the polymorphism of cp genomes.

The two sections (sect. *Nobilia* and sect. *Leontopodium*) were included in subgen. *Leontopodium*, mainly based on plant height, leaves with sheaths or not, and stems being woody or herbaceous (Lin, 1965); however, this division is controversial. First, there were many variations in the height of *Leontopodium* plants. For example, *L. souliei*, belonging to sect. *Leontopodium* was 6–25 cm in height; *L. giraldii* (sect. *Leontopodium*) was 10–28 cm in height; *L. franchetii* belonging to sect. *Nobilia* was 15–50 cm in height; and *L. stracheyi* (sect. *Nobilia*) was 5–60 cm in height (Lin, 1965; Chen et al., 2011). Subsequently, we collated the stem types and found that most species in sect. *Leontopodium* exhibited herbaceous stems, but a few species had woody stems, such as *L. jacotianum*, *L. conglobatum*, and *L. fangingense*; moreover, most species of sect. *Nobilia* has woody stems, but a few species also have herbaceous stems, such as *L. stracheyi*, *L. franchetii*, *L. wilsonii*, *L. sinense*, and *L. andersonii* (Lin, 1965; Chen et al., 2011). In addition, the morphological characteristics of the leaf bases varied within the sections. The leaves of most of the species in sect. *Leontopodium* (other than *L. leontopodioides* and *L. microphyllum*) were amplexicaul or had sheaths, and the leaves of most species in sect. *Nobilia* (other than *L. stracheyi*, *L. franchetii*, *L. artemisiifolium*, and *L. dedekensii*) are not amplexicaul and have no sheaths (Lin, 1965; Chen et al., 2011). The results of our phylogenetic analysis, based on cp genomes, also indicated that sect. *Nobilia* was nested with sect. *Leontopodium*, and did not support the monophyly of the two sections (**Figure 5**). Ma et al. (2022) investigated the achene morphological characteristics of 29 Chinese *Leontopodium* taxa and divided them into two types based on surface ornamentation (reticulate and rippled). Therefore, we matched the main characteristics (achene surface and carpopodium base) of achene reported by Ma et al. (2022) with the cp genome phylogenetic tree but found that species with the same achene characteristics did not cluster together (**Figure 5**). It follows that the previous division of the subgen. *Leontopodium*, based on its morphological characteristics is unreasonable. Although, at the section level, our molecular phylogenetic results were inconsistent with the previous taxonomy system based on morphological characteristics, we found that the characteristics of leaf base, stem types, and carpopodium base had phylogenetic correlation and had potential value in the taxonomic study of *Leontopodium*. In the phylogenetic trees inferred using chloroplast genomes, the subgen. *Leontopodium* was divided into two clades (Clades 1 and 2; **Figure 5** and **Supplementary Figure 1**), with most species in Clade 1 having herbaceous stems, amplexicaul or sheathed leaves, and constricted carpopodium; most species in Clade 2 had woody stems, not amplexicaul and sheathed leaves, and not constricted carpopodium (**Figure 5** and **Supplementary Figure 1**). The phylogenetically conserved pattern of leaf base, stem type, and carpopodium base in *Leontopodium* might be due to greater genetic constraints and/or stabilizing selection pressure favoring stasis of these characters in alpine habitats. Interestingly, similar examples were found in the *Anaphalis* DC. (Asteraceae, Gnaphalieae), a genus with approximately 110 species, and is also the most diversified in the eastern Himalayas and Hengduan Mountains. A recent phylogenetic analysis of *Anaphalis* also suggested a higher degree of homoplasy in the leaf base than in other characters (Nie et al., 2013).

The interspecies relationships of *Leontopodium* are more complex than those of the infrageneric taxonomy system. For example, in the cp genome phylogenetic tree, *L. ochroleucum*, *L. himalayanum*, *L. stracheyi*, *L. leontopodioides*, and *L. franchetii* were clustered together with strong support (BS = 86; PP = 1) (**Figure 5**); however, these five species were dispersed into three main clades in the nrDNA tree (Clades A1, A3, and A5; **Figure 6**). Moreover, they belong to different sections, subsections, and series, based on their morphological characteristics (Handel-Mazzetti, 1928; Lin, 1965). Although the analysis of complete cp genomes allows the clarification of interspecies relationships, it might be insufficient to fully resolve all phylogenetic relationships, especially in rapidly differentiated Asteraceae (Wortley et al., 2005; Petersen et al., 2011; Li et al., 2015; Hatmaker et al., 2020; Kim et al., 2020; Loeuille et al., 2021; Wang et al., 2021). Because the plastome is regarded as a linked single locus due to its uniparental inheritance, multilocus approaches (including mitochondrial genomes and more nuclear genes) are needed to generate abundant and detailed molecular data for systematic classification and evolutionary research. Thus, future studies should analyze additional specimens from wild populations and obtain more extensive genetic and morphological data to verify the taxonomic and phylogenetic identities of *Leontopodium* species.

## 5 Conclusions

In this study, we analyzed the characteristics of 43 cp genomes of *Leontopodium* and its closely related genera. Subsequently, the phylogenetic position of *Leontopodium* and the relationships within this genus were inferred based on complete cp genomes and nrDNA. Finally, together with the morphological characteristics, the relationships within *Leontopodium* were identified and discussed. The cp genomes of *Leontopodium*, *Filago*, and *Gamochaeta* exhibited a typical quadripartite structure, including 85 protein-coding genes, 36–37 tRNA genes, and eight rRNA genes, with a total length of 150,754–151,574 bp. Compared with *Filago* and *Gamochaeta*, *Leontopodium* had longer whole cp genomes and LSC and SSC regions, whereas the length of the IRs was slightly shorter. Genes located at the junctions were well-conserved among the 43 cp genomes. Furthermore, the genic and IR regions were more conserved than the intergenic and SC regions, respectively, which is typical of angiosperm cp genomes. In addition, the cp genome structure of *Leontopodium* exhibited high consistency and was obviously different from those of *Filago* and *Gamochaeta* in some regions, such as *matk*, *trnK (UUU)-rps16*, *petN-psbM*, and *trnE (UUC)-rpoB*. Moreover, we detected 14 hotspots in non-coding regions that could be used as candidate DNA barcodes. All the phylogenetic trees indicated that *Leontopodium* was monophyletic. However, topological trees constructed using cp genomes and nrDNA were incongruent. *Leontopodium* species were divided into three main clades in the cp genome phylogenetic tree and six main clades in the nuclear gene phylogenetic tree. Compared with nrDNA trees, cp trees had higher support values and were more effective for phylogenetic resolution. Except for the subgeneric level, our molecular phylogenetic results were inconsistent with the previous taxonomic system (sections, subsections, and series), which was based on morphological characteristics. Nevertheless, we found that the characteristics of the leaf base, stem types, and carpopodium base were phylogenetically correlated and may have potential value in the taxonomic study of *Leontopodium*. In the phylogenetic trees inferred using chloroplast genomes, the subgen. *Leontopodium* was divided into two clades (Clades 1 and 2): most species in Clade 1 had herbaceous stems, amplexicaul or sheathed leaves, and constricted carpopodium; most species in Clade 2 had woody stems, not amplexicaul and sheathed leaves, and not constricted carpopodium.

## Data availability statement

The datasets presented in this study can be found in online repositories. The names of the repository/repositories and accession number(s) can be found in the article/**Supplementary Material**.

## Author contributions

S-XZ and X-MX conceived of the study. Q-FZ, X-MX, YL, and S-XZ performed fieldwork. X-MX and Q-FZ conducted the laboratory work and wrote the first draft. X-MX, Q-FZ and J-ZS analyzed data. ZW and Z-LW helped improve language. All authors contributed to the article and approved the submitted version.

## Supplementary material

Supplementary Figure 1 BI tree of *Leontopodium*, *Filago*, and *Gamochaeta*, together with *Calendula arvensis* as an outgroup, was inferred from the complete chloroplast genomes.

Supplementary Figure 2 Phylogenetic trees of *Leontopodium* and its closely related genera, together with *Calendula arvensis* as an outgroup, were inferred from coding genes of chloroplast genomes. **(A)** Topology of the ML tree. **(B)** Topology of the BI tree. **(C)** ML tree, with bootstrap values of ML and posterior probabilities of BI shown at each node. Bootstrap values higher than 70 and posterior probabilities higher than 0.90 are indicated on branches. “-” means that the bootstrap value/posterior probability is less than 70/0.90.

Supplementary Figure 3 Phylogenetic trees of *Leontopodium* and its closely related genera, together with *Calendula arvensis* as an outgroup, were inferred from the ITS sequences. **(A)** Topology of the ML tree. **(B)** Topology of the BI tree. **(C)** ML tree, with bootstrap values of ML and posterior probabilities of BI shown at each node. Bootstrap values higher than 70 and posterior probabilities higher than 0.90 are indicated on branches. “-” means that the bootstrap value/posterior probability is less than 70/0.90.

Supplementary Figure 4 Phylogenetic trees of *Leontopodium* and its closely related genera, together with *Calendula arvensis* as an outgroup, were inferred from the ETS sequences. **(A)** Topology of the ML tree. **(B)** Topology of the BI tree. **(C)** ML tree, with bootstrap values of ML and posterior probabilities of BI shown at each node. Bootstrap values higher than 70 and posterior probabilities higher than 0.90 are indicated on branches. “-” means that the bootstrap value/posterior probability is less than 70/0.90.

Supplementary Figure 5 Phylogenetic trees of *Leontopodium* and its closely related genera, together with *Calendula arvensis* as an outgroup, were inferred from the concatenated sequences of ITS and ETS. **(A)** Topology of the ML tree. **(B)** Topology of the BI tree. **(C)** ML tree, with bootstrap values of ML and posterior probabilities of BI shown at each node. Bootstrap values higher than 70 and posterior probabilities higher than 0.90 are indicated on branches. “-” means that the bootstrap value/posterior probability is less than 70/0.90.
