# Supplementary figures and images for: Phylogeny of Leontopodium (Asteraceae) in China—with a reference to plastid genome and nuclear ribosomal DNA
Source: Front Plant Sci. 2023 Jul 31;14:1163065. doi: 10.3389/fpls.2023.1163065 (PMC10425225; doi:10.3389/fpls.2023.1163065)

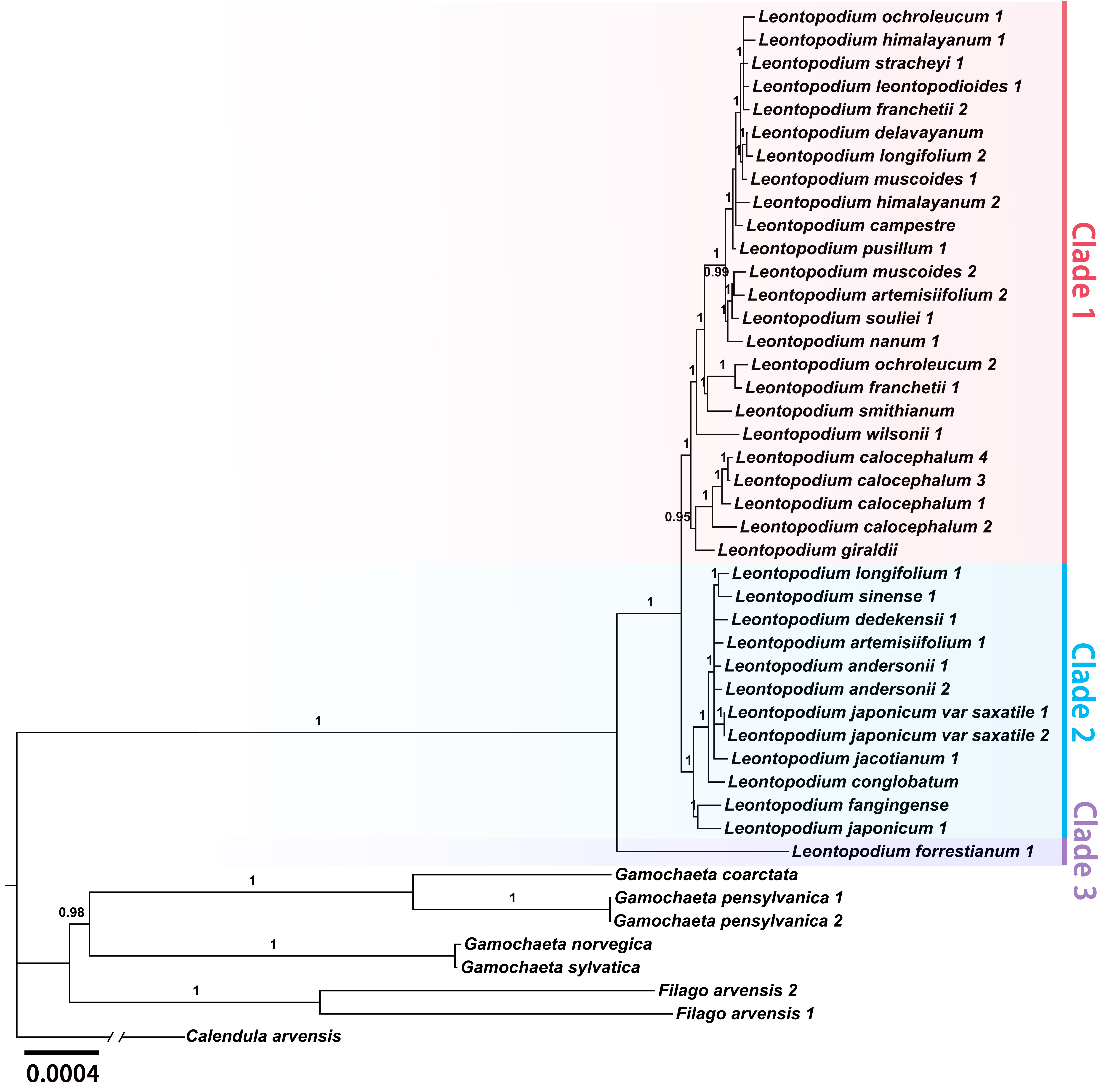

Supplement: Supplementary Figure 1 — BI tree of Leontopodium, Filago, and Gamochaeta, together with Calendula arvensis as an outgroup, was inferred from the complete chloroplast genomes. [file Image_1.jpeg]

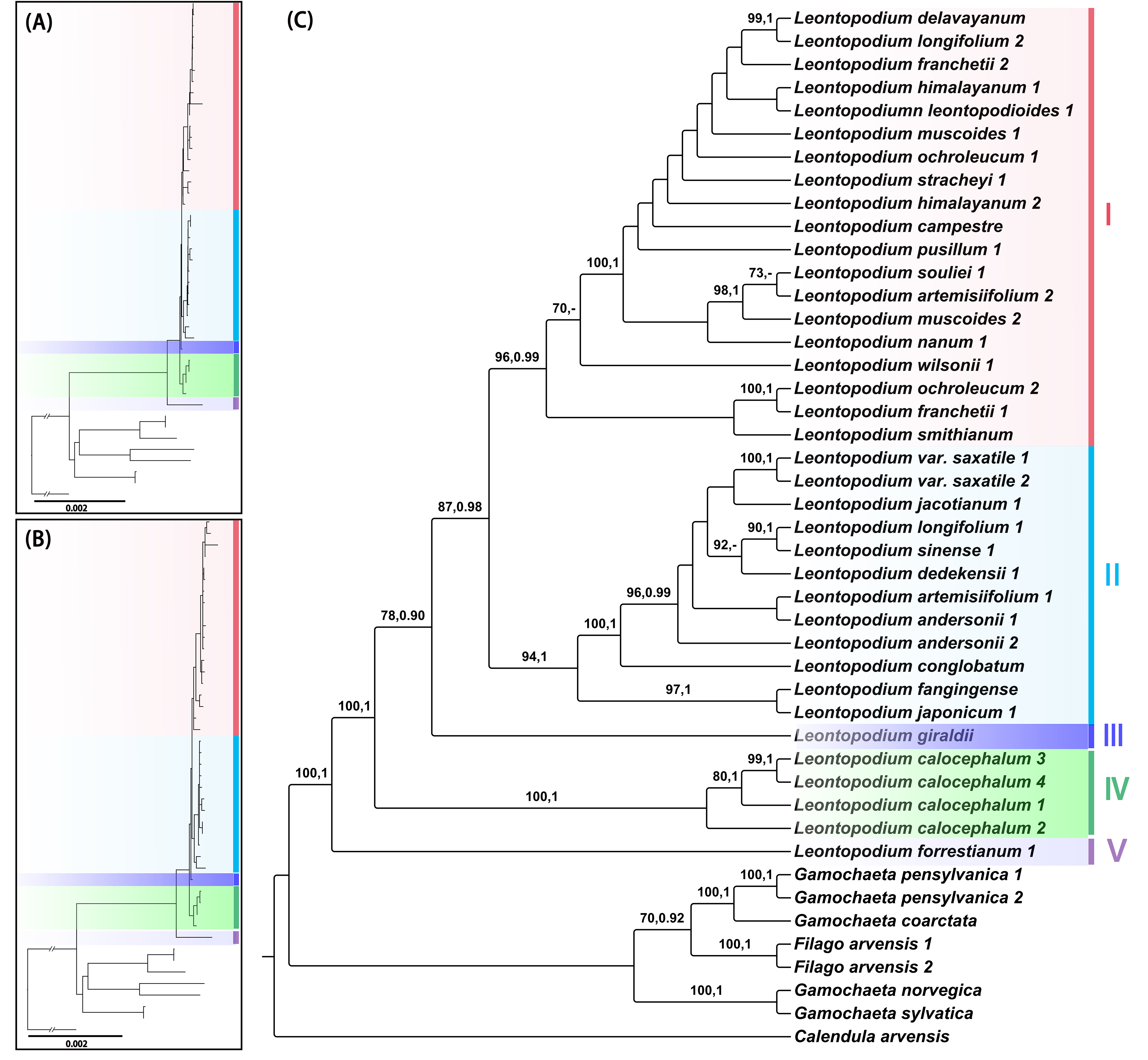

Supplement: Supplementary Figure 2 — Phylogenetic trees of Leontopodium and its closely related genera, together with Calendula arvensis as an outgroup, were inferred from coding genes of chloroplast genomes. (A) Topology of the ML tree. (B) Topology of the BI tree. (C) ML tree, with bootstrap values of ML and posterior probabilities of BI shown at each node. Bootstrap values higher than 70 and posterior probabilities higher than 0.90 are indicated on branches. “-” means that the bootstrap value/posterior probability is less than 70/0.90. [file Image_2.jpg]

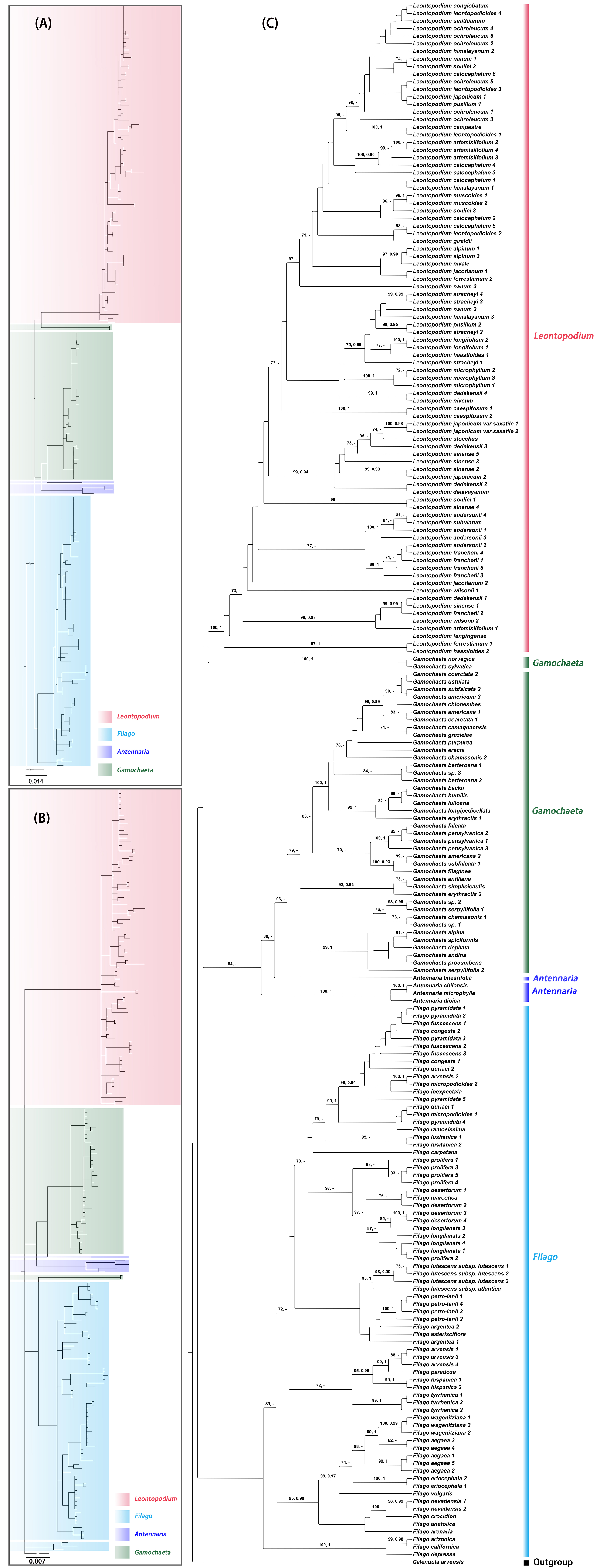

Supplement: Supplementary Figure 3 — Phylogenetic trees of Leontopodium and its closely related genera, together with Calendula arvensis as an outgroup, were inferred from the ITS sequences. (A) Topology of the ML tree. (B) Topology of the BI tree. (C) ML tree, with bootstrap values of ML and posterior probabilities of BI shown at each node. Bootstrap values higher than 70 and posterior probabilities higher than 0.90 are indicated on branches. “-” means that the bootstrap value/posterior probability is less than 70/0.90. [file Image_3.jpg]

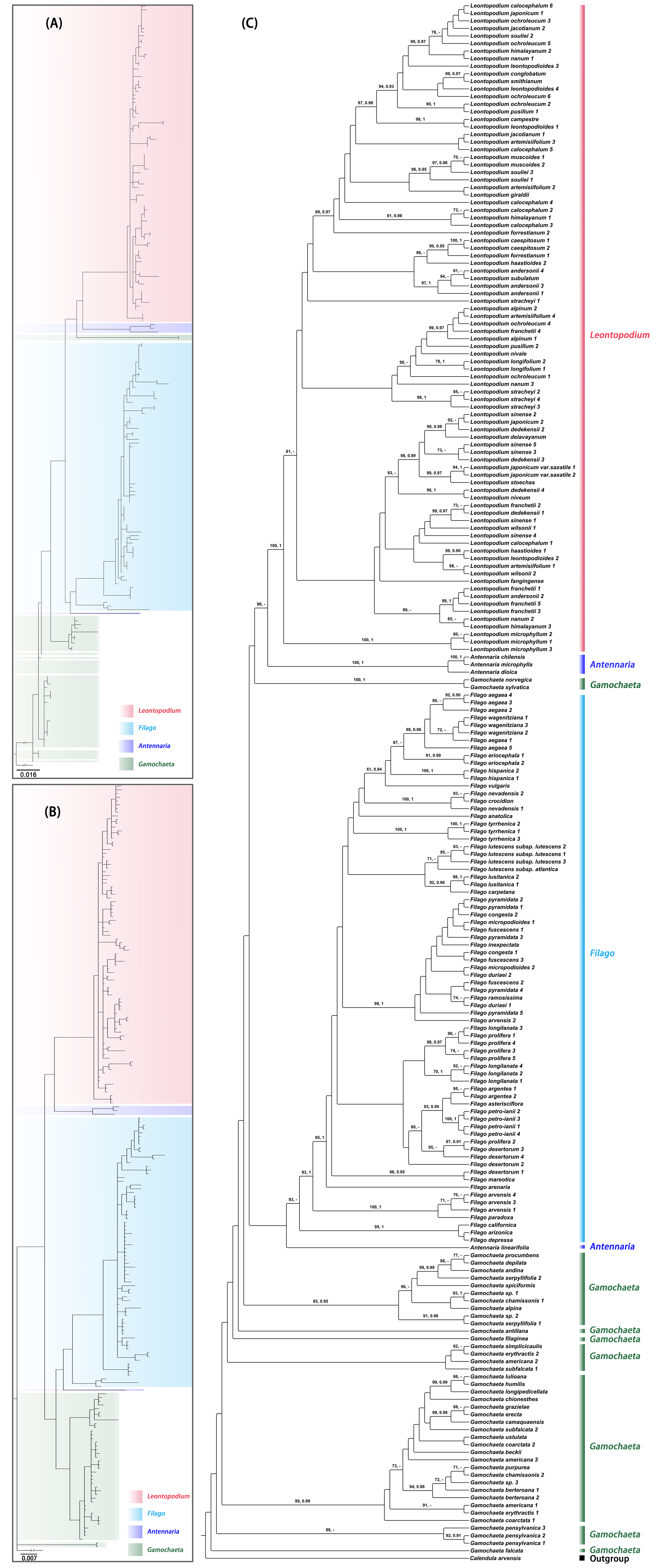

Supplement: Supplementary Figure 4 — Phylogenetic trees of Leontopodium and its closely related genera, together with Calendula arvensis as an outgroup, were inferred from the ETS sequences. (A) Topology of the ML tree. (B) Topology of the BI tree. (C) ML tree, with bootstrap values of ML and posterior probabilities of BI shown at each node. Bootstrap values higher than 70 and posterior probabilities higher than 0.90 are indicated on branches. “-” means that the bootstrap value/posterior probability is less than 70/0.90. [file Image_4.jpg]

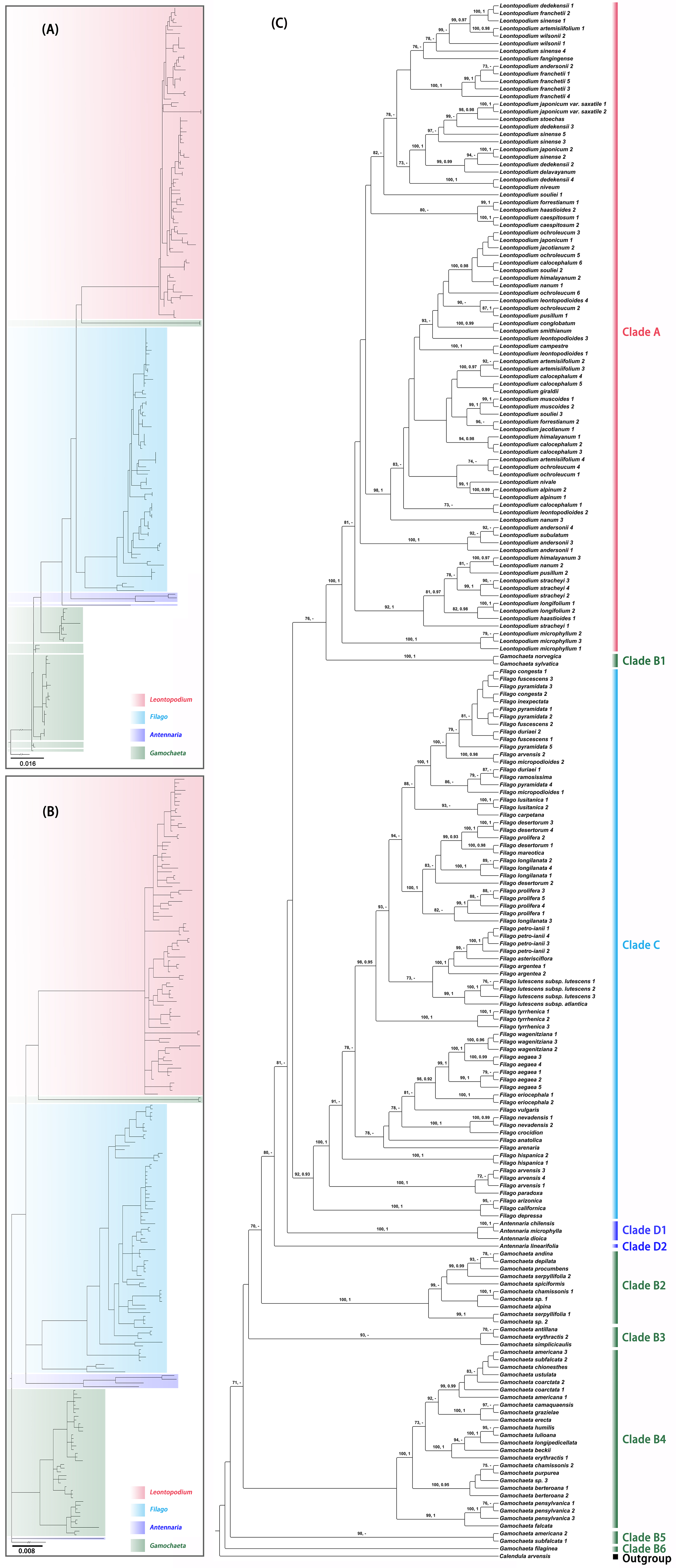

Supplement: Supplementary Figure 5 — Phylogenetic trees of Leontopodium and its closely related genera, together with Calendula arvensis as an outgroup, were inferred from the concatenated sequences of ITS and ETS. (A) Topology of the ML tree. (B) Topology of the BI tree. (C) ML tree, with bootstrap values of ML and posterior probabilities of BI shown at each node. Bootstrap values higher than 70 and posterior probabilities higher than 0.90 are indicated on branches. “-” means that the bootstrap value/posterior probability is less than 70/0.90. [file Image_5.jpg]
