# Supplementary material for: Phylogeny of Leontopodium (Asteraceae) in China—with a reference to plastid genome and nuclear ribosomal DNA
Source: Front Plant Sci. 2023 Jul 31;14:1163065. doi: 10.3389/fpls.2023.1163065 (PMC10425225; doi:10.3389/fpls.2023.1163065)
Supplement: Supplementary file 6 [file Table_1.docx]

**Supplementary Table 1 |** Accession numbers of ITS and ETS from Genbank

| Taxa | ITS | ETS |
| --- | --- | --- |
| *Antennaria chilensis* | KM091395 | KM091373 |
| *Antennaria dioica* | FN645833 | FN645610 |
| *Antennaria linearifolia* | MF118846 | MF118814 |
| *Antennaria microphylla* | HM244731 | HM450870 |
| *Filago aegaea 1* | HG918219 | HG918234 |
| *Filago aegaea 2* | HG918220 | HG918235 |
| *Filago aegaea 3* | HG918221 | HG918236 |
| *Filago aegaea 4* | HG918222 | HG918237 |
| *Filago aegaea 5* | FN645865 | FN645602 |
| *Filago anatolica* | FN645857 | FN645598 |
| *Filago arenaria* | FN645887 | FN645597 |
| *Filago argentea 1* | FN645859 | FN645569 |
| *Filago argentea 2* | FN645860 | FN645570 |
| *Filago arizonica* | FN645839 | FN645615 |
| *Filago arvensis 3* | FN645886 | FN645606 |
| *Filago arvensis 4* | FN645885 | FN645605 |
| *Filago asterisciflora* | FN645861 | FN645571 |
| *Filago californica* | FN645840 | FN645616 |
| *Filago carpetana* | FN645858 | FN645568 |
| *Filago congesta 1* | FN645871 | FN645578 |
| *Filago congesta 2* | FN645848 | FN645577 |
| *Filago crocidion* | FN645864 | FN645601 |
| *Filago depressa* | FN645841 | FN645617 |
| *Filago desertorum 1* | FN645875 | FN645592 |
| *Filago desertorum 2* | FN645874 | FN645591 |
| *Filago desertorum 3* | KY824542 | KY824529 |
| *Filago desertorum 4* | KY824533 | KY824520 |
| *Filago duriaei 1* | FN645881 | FN645587 |
| *Filago duriaei 2* | FN645849 | FN645586 |
| *Filago eriocephala 1* | MF118852 | MF118819 |
| *Filago eriocephala 2* | FN645884 | FN645603 |
| *Filago fuscescens 1* | FN645847 | FN645585 |
| *Filago fuscescens 2* | FN645846 | FN645580 |
| *Filago fuscescens 3* | FN645845 | FN645579 |
| *Filago hispanica 1* | FN645856 | FN645567 |
| *Filago hispanica 2* | FN645855 | FN645566 |
| *Filago inexpectata* | FN645852 | FN645584 |
| *Filago longilanata 1* | KY824536 | KY824523 |
| *Filago longilanata 2* | KY824540 | KY824527 |
| *Filago longilanata 3* | KY824534 | KY824521 |
| *Filago longilanata 4* | KY824539 | KY824526 |
| *Filago lusitanica 1* | FN645867 | FN645573 |
| *Filago lusitanica 2* | FN645866 | FN645572 |
| *Filago lutescens* subsp. *atlantica* | FN645877 | FN645595 |
| *Filago lutescens* subsp*. lutescens 1* | FN645882 | FN645596 |
| *Filago lutescens* subsp*. lutescens 2* | FN645883 | FN645581 |
| *Filago lutescens* subsp*. lutescens 3* | FN645876 | FN645594 |
| *Filago mareotica* | FN645879 | FN645593 |
| *Filago micropodioides 1* | FN645850 | FN645582 |
| *Filago micropodioides 2* | FN645851 | FN645583 |
| *Filago nevadensis 1* | FN645862 | FN645599 |
| *Filago nevadensis 2* | FN645863 | FN645600 |
| *Filago paradoxa* | FN645889 | FN645607 |
| *Filago petro-ianii 1* | HG918226 | HG918241 |
| *Filago petro-ianii 2* | HG918225 | HG918240 |
| *Filago petro-ianii 3* | HG918223 | HG918238 |
| *Filago petro-ianii 4* | HG918224 | HG918239 |
| *Filago prolifera 1* | KY824538 | KY824525 |
| *Filago prolifera 2* | KY824537 | KY824524 |
| *Filago prolifera 3* | KY824541 | KY824528 |
| *Filago prolifera 4* | KY824543 | KY824530 |
| *Filago prolifera 5* | KY824532 | KY824519 |
| *Filago pyramidata 1* | MF118853 | MF118820 |
| *Filago pyramidata 2* | FN645872 | FN645588 |
| *Filago pyramidata 3* | FN645873 | FN645590 |
| *Filago pyramidata 4* | AY445231 | FN645589 |
| *Filago pyramidata 5* | KY824531 | KY824518 |
| *Filago ramosissima* | FN645880 | FN645563 |
| *Filago tyrrhenica 1* | HG918227 | HG918242 |
| *Filago tyrrhenica 2* | HG918228 | HG918243 |
| *Filago tyrrhenica 3* | HG918229 | HG918244 |
| *Filago vulgaris* | FN645878 | FN645604 |
| *Filago wagenitziana 1* | HG918231 | HG918246 |
| *Filago wagenitziana 2* | HG918232 | HG918247 |
| *Filago wagenitziana 3* | HG918233 | HG918248 |
| *Gamochaeta alpina* | MF118862 | MF118829 |
| *Gamochaeta americana 1* | KT865472 | KT865270 |
| *Gamochaeta americana 2* | MF118854 | MF118821 |
| *Gamochaeta americana 3* | KM091411 | KM091382 |
| *Gamochaeta andina* | KX077990 | KX078018 |
| *Gamochaeta antillana* | KX077991 | KX078019 |
| *Gamochaeta beckii* | KX077992 | KX078021 |
| *Gamochaeta berteroana 1* | MF118856 | MF118823 |
| *Gamochaeta berteroana 2* | MF118855 | MF118822 |
| *Gamochaeta camaquaensis* | KX077993 | KX078023 |
| *Gamochaeta chamissonis 1* | MF118857 | MF118824 |
| *Gamochaeta chamissonis 2* | KX077994 | KX078024 |
| *Gamochaeta chionesthes* | KX077995 | KX078025 |
| *Gamochaeta coarctata 1* | KT865473 | KT865271 |
| *Gamochaeta coarctata 2* | KX077996 | KX078026 |
| *Gamochaeta depilata* | KX077997 | KX078027 |
| *Gamochaeta erecta* | KX077998 | KX078029 |
| *Gamochaeta erythractis 1* | KT865474 | KT865272 |
| *Gamochaeta erythractis 2* | KX077999 | KX078030 |
| *Gamochaeta falcata* | KT865475 | KT865273 |
| *Gamochaeta filaginea* | KT865476 | KT865274 |
| *Gamochaeta grazielae* | KX078001 | KX078033 |
| *Gamochaeta humilis* | KX078002 | KX078034 |
| *Gamochaeta longipedicellata* | KM091410 | KM091381 |
| *Gamochaeta lulioana* | KX078003 | KX078035 |
| *Gamochaeta pensylvanica 3* | KX078004 | KX078037 |
| *Gamochaeta procumbens* | KX078005 | KX078038 |
| *Gamochaeta purpurea* | KX078006 | KX078039 |
| *Gamochaeta serpyllifolia 1* | MF118858 | MF118825 |
| *Gamochaeta serpyllifolia 2* | KM091409 | KM091380 |
| *Gamochaeta simplicicaulis* | KX078007 | KX078040 |
| *Gamochaeta sp. 1* | MF118859 | MF118827 |
| *Gamochaeta sp. 2* | MF118860 | MF118828 |
| *Gamochaeta sp. 3* | MF118861 | MF118826 |
| *Gamochaeta spiciformis* | KX078011 | KX078042 |
| *Gamochaeta subfalcata 1* | FN645834 | FN645557 |
| *Gamochaeta subfalcata 2* | KX078009 | KX078044 |
| *Gamochaeta ustulata* | KX078012 | KX078045 |
| *Leontopodium alpinum 1* | FJ639913 | FJ639981 |
| *Leontopodium alpinum 2* | KT865530 | KT865346 |
| *Leontopodium andersonii 3* | FJ639922 | FJ640006 |
| *Leontopodium andersonii 4* | KT865531 | KT865347 |
| *Leontopodium artemisiifolium 3* | FJ639924 | FJ640009 |
| *Leontopodium artemisiifolium 4* | FJ639925 | FJ639982 |
| *Leontopodium caespitosum 1* | FJ639926 | FJ640010 |
| *Leontopodium caespitosum 2* | FJ639927 | FJ640011 |
| *Leontopodium calocephalum 5* | FJ639929 | FJ640012 |
| *Leontopodium calocephalum 6* | FJ639928 | FJ639994 |
| *Leontopodium dedekensii 2* | FJ639932 | FJ640002 |
| *Leontopodium dedekensii 3* | FJ639930 | FJ640001 |
| *Leontopodium dedekensii 4* | KT865532 | KT865348 |
| *Leontopodium forrestianum 2* | KT865533 | KT865350 |
| *Leontopodium franchetii 3* | FJ639935 | FJ640003 |
| *Leontopodium franchetii 4* | FJ639934 | FJ639983 |
| *Leontopodium franchetii 5* | KT865534 | KT865351 |
| *Leontopodium haastioides 1* | FJ639937 | FJ640013 |
| *Leontopodium haastioides 2* | KT865535 | KT865352 |
| *Leontopodium himalayanum 3* | FJ639938 | FJ640004 |
| *Leontopodium jacotianum 2* | FJ639940 | FJ639995 |
| *Leontopodium japonicum 2* | KT865536 | KT865353 |
| *Leontopodium leontopodioides 2* | FJ639944 | FJ640014 |
| *Leontopodium leontopodioides 3* | KT865537 | KT865354 |
| *Leontopodium leontopodioides 4* | KT865538 | KT865355 |
| *Leontopodium microphyllum 1* | FJ639946 | FJ640015 |
| *Leontopodium microphyllum 2* | FJ639947 | FJ640016 |
| *Leontopodium microphyllum 3* | KT865539 | KT865356 |
| *Leontopodium nanum 2* | FJ639951 | FJ640005 |
| *Leontopodium nanum 3* | KT865540 | KT865358 |
| *Leontopodium nivale* | FJ639950 | FJ640017 |
| *Leontopodium niveum* | KT865541 | KT865359 |
| *Leontopodium ochroleucum 3* | FJ639954 | FJ639997 |
| *Leontopodium ochroleucum 4* | FJ639953 | FJ639990 |
| *Leontopodium ochroleucum 5* | FJ639952 | FJ639996 |
| *Leontopodium ochroleucum 6* | KT865542 | KT865360 |
| *Leontopodium pusillum 2* | FJ639955 | FJ640018 |
| *Leontopodium sinense 2* | FJ639957 | FJ640000 |
| *Leontopodium sinense 3* | FJ639958 | FJ639999 |
| *Leontopodium sinense 4* | KT865543 | KT865361 |
| *Leontopodium sinense 5* | JQ895512 | JQ895375 |
| *Leontopodium souliei 2* | FJ639959 | FJ639998 |
| *Leontopodium souliei 3* | KT865544 | KT865362 |
| *Leontopodium stoechas* | KT865545 | KT865363 |
| *Leontopodium stracheyi 2* | FJ639960 | FJ640020 |
| *Leontopodium stracheyi 3* | KT865546 | KT865364 |
| *Leontopodium stracheyi 4* | FJ639961 | FJ640022 |
| *Leontopodium subulatum* | KT865547 | KT865365 |
| *Leontopodium wilsonii 2* | JQ895513 | JQ895376 |
