# Supplementary material for: Phylogeny of Leontopodium (Asteraceae) in China—with a reference to plastid genome and nuclear ribosomal DNA
Source: Front Plant Sci. 2023 Jul 31;14:1163065. doi: 10.3389/fpls.2023.1163065 (PMC10425225; doi:10.3389/fpls.2023.1163065)
Supplement: Supplementary file 7 [file Table_2.docx]

**Supplementary Table 2 |** The length, aligned length, number of haplotypes (H), haplotype (gene) diversity (Hd), nucleotide diversity (Pi), total number of mutation (Eta), singleton variable sites and parsimony informative sites of conding regions (aligned length>200 bp)*.*

| Region | Length (bp) | Aligned length (bp) | Number of Haplotypes (H) | Haplotype (gene) diversity (Hd) | Nucleotide diversity (Pi) | Total number of mutation (Eta) | Singleton variable sites | Parsimony informative sites |
| --- | --- | --- | --- | --- | --- | --- | --- | --- |
| accD | 1503-1508 | 1518 | 11 | 0.545 | 0.00168 | 23 | 7 | 16 |
| atpA | 1527 | 1527 | 7 | 0.588 | 0.00190 | 20 | 5 | 15 |
| atpB | 1479-1488 | 1488 | 7 | 0.410 | 0.00231 | 27 | 3 | 22 |
| atpE | 402 | 402 | 3 | 0.286 | 0.00073 | 2 | 1 | 1 |
| atpF | 555 | 555 | 7 | 0.339 | 0.00177 | 8 | 2 | 6 |
| atpH | 246 | 246 | 3 | 0.135 | 0.00075 | 3 | 2 | 1 |
| atpI | 744 | 744 | 6 | 0.440 | 0.00181 | 9 | 3 | 6 |
| ccsA | 948 | 948 | 9 | 0.773 | 0.00190 | 9 | 1 | 8 |
| cemA | 690 | 690 | 5 | 0.333 | 0.00203 | 7 | 1 | 6 |
| clpP | 591 | 591 | 6 | 0.406 | 0.00076 | 5 | 2 | 3 |
| infA | 234 | 234 | 3 | 0.476 | 0.00215 | 2 | 0 | 2 |
| matK | 1512-1521 | 1521 | 12 | 0.760 | 0.00397 | 49 | 14 | 35 |
| ndhA | 1092 | 1092 | 9 | 0.447 | 0.00125 | 15 | 7 | 8 |
| ndhB | 1533 | 1533 | 3 | 0.092 | 0.00015 | 3 | 1 | 2 |
| ndhC | 363 | 363 | 3 | 0.135 | 0.00038 | 2 | 1 | 1 |
| ndhD | 1503 | 1503 | 10 | 0.658 | 0.00145 | 19 | 9 | 10 |
| ndhE | 306 | 306 | 4 | 0.178 | 0.00060 | 3 | 2 | 1 |
| ndhF | 2226-2277 | 2283 | 15 | 0.795 | 0.00294 | 57 | 20 | 37 |
| ndhG | 531 | 531 | 7 | 0.339 | 0.00186 | 11 | 4 | 7 |
| ndhH | 1182 | 1182 | 11 | 0.601 | 0.00201 | 18 | 7 | 11 |
| ndhI | 501 | 501 | 3 | 0.135 | 0.00027 | 2 | 1 | 1 |
| ndhJ | 477 | 477 | 7 | 0.504 | 0.00293 | 9 | 2 | 7 |
| ndhK | 678 | 678 | 5 | 0.296 | 0.00100 | 6 | 3 | 2 |
| petA | 957 | 957 | 7 | 0.339 | 0.00094 | 17 | 6 | 11 |
| petB | 648 | 648 | 2 | 0.279 | 0.00043 | 1 | 0 | 1 |
| petD | 483 | 483 | 6 | 0.300 | 0.00145 | 6 | 1 | 5 |
| psaA | 2253 | 2253 | 7 | 0.339 | 0.00094 | 17 | 6 | 11 |
| psaB | 1698-2205 | 2205 | 7 | 0.339 | 0.00131 | 28 | 13 | 13 |
| psaC | 246 | 246 | 2 | 0.246 | 0.00100 | 1 | 0 | 1 |
| psbA | 1062 | 1062 | 5 | 0.260 | 0.00086 | 7 | 1 | 6 |
| psbB | 1527 | 1527 | 9 | 0.447 | 0.00143 | 21 | 7 | 14 |
| psbC | 1422 | 1422 | 7 | 0.588 | 0.00107 | 11 | 3 | 8 |
| psbD | 1062 | 1062 | 5 | 0.590 | 0.00093 | 7 | 2 | 5 |
| psbE | 252 | 252 | 1 | 0.000 | 0.00000 | 0 | 0 | 0 |
| psbH | 219-222 | 222 | 4 | 0.258 | 0.00216 | 4 | 1 | 3 |
| rbcL | 1455-1458 | 1458 | 13 | 0.823 | 0.00464 | 40 | 9 | 29 |
| rpl14 | 369 | 369 | 5 | 0.298 | 0.00098 | 5 | 3 | 2 |
| rpl16 | 408 | 408 | 6 | 0.338 | 0.00172 | 7 | 2 | 5 |
| rpl2 | 828 | 828 | 5 | 0.220 | 0.00028 | 4 | 3 | 1 |
| rpl20 | 381 | 381 | 3 | 0.251 | 0.00196 | 4 | 1 | 3 |
| rpl22 | 453-462 | 462 | 3 | 0.092 | 0.00030 | 2 | 1 | 1 |
| rpl23 | 282 | 282 | 3 | 0.286 | 0.00186 | 2 | 0 | 2 |
| rpl33 | 201 | 201 | 3 | 0.255 | 0.00167 | 2 | 0 | 2 |
| rpoA | 1008 | 1008 | 8 | 0.377 | 0.00083 | 10 | 5 | 5 |
| rpoB | 3183 | 3183 | 7 | 0.375 | 0.00112 | 26 | 6 | 19 |
| rpoC1 | 2085 | 2085 | 12 | 0.516 | 0.00134 | 25 | 12 | 13 |
| rpoC2 | 4125-4134 | 4134 | 13 | 0.631 | 0.00145 | 50 | 19 | 31 |
| rps11 | 411 | 411 | 4 | 0.219 | 0.00056 | 3 | 1 | 2 |
| rps12 | 357 | 357 | 1 | 0.000 | 0.00000 | 0 | 0 | 0 |
| rps14 | 303 | 303 | 5 | 0.299 | 0.00172 | 5 | 2 | 3 |
| rps15 | 279 | 279 | 5 | 0.296 | 0.00263 | 7 | 4 | 3 |
| rps16 | 267 | 267 | 5 | 0.298 | 0.00192 | 4 | 2 | 2 |
| rps18 | 306 | 306 | 3 | 0.177 | 0.00059 | 2 | 0 | 2 |
| rps19 | 279 | 279 | 6 | 0.501 | 0.00206 | 5 | 4 | 1 |
| rps2 | 711 | 711 | 4 | 0.291 | 0.00089 | 5 | 3 | 2 |
| rps3 | 657 | 657 | 5 | 0.220 | 0.00070 | 8 | 5 | 2 |
| rps4 | 606 | 606 | 6 | 0.338 | 0.00101 | 7 | 4 | 3 |
| rps7 | 468 | 468 | 2 | 0.091 | 0.00019 | 1 | 0 | 1 |
| rps8 | 405 | 405 | 3 | 0.177 | 0.00067 | 3 | 0 | 3 |
| rrn16S | 1491 | 1491 | 3 | 0.092 | 0.00012 | 3 | 1 | 1 |
| rrn23S | 2805 | 2805 | 6 | 0.626 | 0.00047 | 10 | 3 | 7 |
| ycf1 | 5112-5133 | 5145 | 29 | 0.970 | 0.00337 | 145 | 56 | 84 |
| ycf2 | 6648-6654 | 6654 | 7 | 0.339 | 0.00014 | 8 | 3 | 5 |
| ycf3 | 507 | 507 | 4 | 0.256 | 0.00267 | 11 | 6 | 5 |
| ycf4 | 555 | 555 | 4 | 0.219 | 0.00041 | 3 | 1 | 2 |
