# Supplementary material for: Phylogeny of Leontopodium (Asteraceae) in China—with a reference to plastid genome and nuclear ribosomal DNA
Source: Front Plant Sci. 2023 Jul 31;14:1163065. doi: 10.3389/fpls.2023.1163065 (PMC10425225; doi:10.3389/fpls.2023.1163065)
Supplement: Supplementary file 8 [file Table_3.docx]

**Supplementary Table 3 |** The length, aligned length, number of haplotypes (H), haplotype (gene) diversity (Hd), nucleotide diversity (Pi), total number of mutation (Eta), singleton variable sites and parsimony informative sites of non-coding regions (aligned length>200 bp)*.*

| Region | Length (bp) | Aligned length (bp) | Number of Haplotypes (H) | Haplotype (gene) diversity (Hd) | Nucleotide diversity (Pi) | Total number of mutation (Eta) | Singleton variable sites | Parsimony informative sites |
| --- | --- | --- | --- | --- | --- | --- | --- | --- |
| accD-psaI | 813-856 | 880 | 10 | 0.449 | 0.00318 | 27 | 14 | 12 |
| atpB-rbcL | 739-745 | 750 | 9 | 0.413 | 0.00173 | 13 | 5 | 8 |
| atpF intron | 711 | 711 | 5 | 0.260 | 0.00102 | 9 | 2 | 7 |
| atpH-atpF | 355-408 | 411 | 7 | 0.372 | 0.00572 | 16 | 7 | 7 |
| atpI-atpH | 1104-1128 | 1145 | 10 | 0.449 | 0.00308 | 26 | 9 | 17 |
| cemA-petA | 226 | 226 | 3 | 0.092 | 0.00062 | 3 | 3 | 0 |
| clpP-psbB | 458-468 | 477 | 7 | 0.548 | 0.00275 | 9 | 4 | 5 |
| clpP intron 1 | 801-853 | 873 | 9 | 0.691 | 0.00362 | 23 | 8 | 14 |
| clpP intron 2 | 619-630 | 639 | 7 | 0.472 | 0.00275 | 12 | 4 | 8 |
| matK-trnK(UUU) | 733-750 | 753 | 9 | 0.413 | 0.00174 | 15 | 10 | 5 |
| ndhA intron | 1033-1042 | 1046 | 10 | 0.571 | 0.00355 | 36 | 15 | 20 |
| ndhB intron | 670 | 670 | 3 | 0.092 | 0.00014 | 2 | 2 | 0 |
| ndhB-rps7 | 289 | 289 | 2 | 0.047 | 0.00016 | 1 | 1 | 0 |
| ndhC-trnV(UAC) | 1072-1201 | 1226 | 13 | 0.788 | 0.00464 | 40 | 20 | 19 |
| ndhD-ccsA | 258-268 | 275 | 6 | 0.300 | 0.00619 | 16 | 3 | 11 |
| ndhE-psaC | 254-256 | 256 | 4 | 0.256 | 0.00556 | 8 | 2 | 6 |
| ndhF-trnN(GUU) | 793-926 | 926 | 5 | 0.623 | 0.00119 | 5 | 1 | 4 |
| ndhG-ndhE | 219 | 219 | 5 | 0.299 | 0.00441 | 6 | 1 | 5 |
| ndhI-ndhG | 319-326 | 328 | 8 | 0.377 | 0.00701 | 17 | 3 | 14 |
| petA-psbJ | 763-771 | 784 | 10 | 0.677 | 0.00941 | 34 | 4 | 30 |
| petB intron | 757-770 | 777 | 9 | 0.413 | 0.00320 | 16 | 4 | 11 |
| petD intron | 698-707 | 707 | 7 | 0.301 | 0.00312 | 21 | 7 | 13 |
| petD-rpoA | 198-217 | 248 | 5 | 0.260 | 0.00535 | 8 | 3 | 5 |
| petL-petG | 165-228 | 228 | 5 | 0.299 | 0.00519 | 7 | 2 | 5 |
| petN-psbM | 405-541 | 553 | 11 | 0.748 | 0.01433 | 35 | 7 | 27 |
| psaA-ycf3 | 743-766 | 766 | 11 | 0.546 | 0.00390 | 27 | 9 | 17 |
| psaI-ycf4 | 393-411 | 411 | 7 | 0.605 | 0.00367 | 8 | 3 | 5 |
| psaJ-rpl33 | 437-439 | 439 | 6 | 0.300 | 0.00228 | 9 | 3 | 6 |
| psbA-trnK(UUU) | 221-227 | 227 | 6 | 0.334 | 0.00579 | 11 | 7 | 4 |
| psbB-psbT | 183-200 | 202 | 6 | 0.337 | 0.00517 | 7 | 2 | 5 |
| psbC-trnS(UGA) | 223-242 | 242 | 8 | 0.667 | 0.00550 | 9 | 5 | 4 |
| psbE-petL | 1253-1288 | 1300 | 10 | 0.730 | 0.00387 | 38 | 14 | 23 |
| psbK-psbI | 409-417 | 417 | 7 | 0.339 | 0.00402 | 11 | 3 | 8 |
| psbM-trnD(GUC) | 569-660 | 662 | 7 | 0.339 | 0.00425 | 19 | 9 | 10 |
| psbZ-trnG(GCC) | 303-318 | 325 | 8 | 0.551 | 0.00248 | 7 | 5 | 2 |
| rbcL-accD | 475-502 | 524 | 7 | 0.339 | 0.00438 | 14 | 3 | 11 |
| rpl16 intron | 958-982 | 990 | 14 | 0.608 | 0.00249 | 25 | 11 | 13 |
| rpl20-rps12 | 733-751 | 752 | 7 | 0.375 | 0.00162 | 9 | 3 | 6 |
| rpl2 intron | 662 | 666 | 1 | 0.000 | 0.00000 | 0 | 0 | 0 |
| rpl32-ndhF | 726-1025 | 1046 | 10 | 0.719 | 0.00728 | 37 | 9 | 28 |
| rpoC1 intron | 737-741 | 741 | 11 | 0.483 | 0.00263 | 19 | 10 | 8 |
| rpoC2-rps2 | 239-250 | 251 | 8 | 0.377 | 0.00520 | 11 | 5 | 6 |
| rps12-trnV(GAC) | 1640-1852 | 1858 | 9 | 0.413 | 0.00052 | 11 | 7 | 4 |
| rps14-psaB | 133-632 | 632 | 2 | 0.246 | 0.00185 | 1 | 0 | 1 |
| rps16-trnQ(UUG) | 919-1053 | 1095 | 13 | 0.750 | 0.00504 | 41 | 15 | 25 |
| rps16 intron | 844-856 | 868 | 8 | 0.377 | 0.00354 | 33 | 9 | 20 |
| rps18-rpl20 | 251-252 | 252 | 7 | 0.372 | 0.00306 | 7 | 4 | 3 |
| rps2-atpI | 215 | 215 | 4 | 0.295 | 0.00293 | 4 | 1 | 3 |
| rps4-trnT(UGU) | 360-397 | 405 | 4 | 0.295 | 0.00589 | 10 | 1 | 9 |
| rps7-rps12 | 603 | 603 | 1 | 0.000 | 0.00000 | 0 | 0 | 0 |
| rrn16S-trnI(GAU) | 292 | 292 | 3 | 0.092 | 0.00048 | 3 | 3 | 0 |
| rrn4.5S-rrn5S | 244-245 | 245 | 1 | 0.000 | 0.00000 | 0 | 0 | 0 |
| rrn5S-trnR(ACG) | 251-253 | 253 | 2 | 0.246 | 0.00098 | 1 | 0 | 1 |
| trnA(UGC) intron | 821 | 821 | 1 | 0.000 | 0.00000 | 0 | 0 | 0 |
| trnC(GCA)-petN | 360-382 | 385 | 6 | 0.300 | 0.00567 | 18 | 5 | 11 |
| trnE(UUC)-rpoB | 760-903 | 931 | 10 | 0.713 | 0.00597 | 29 | 9 | 19 |
| trnF(GAA)-ndhJ | 393-588 | 588 | 8 | 0.478 | 0.00306 | 11 | 1 | 10 |
| trnG(GCC)-trnfM(CAU) | 216-239 | 243 | 6 | 0.267 | 0.00582 | 16 | 8 | 8 |
| trnG(UCC)-trnT(GGU) | 257-376 | 428 | 4 | 0.271 | 0.00581 | 11 | 0 | 11 |
| trnG(UCC) intron | 696-712 | 713 | 7 | 0.375 | 0.00193 | 12 | 3 | 8 |
| trnH(GUG)-psbA | 342-396 | 410 | 8 | 0.616 | 0.02102 | 51 | 11 | 37 |
| trnI(GAU) intron | 779-780 | 780 | 2 | 0.047 | 0.00006 | 1 | 1 | 0 |
| trnK(UUU)-rps16 | 781-808 | 822 | 9 | 0.695 | 0.00605 | 39 | 13 | 25 |
| trnK(UUU)-matK | 273-306 | 311 | 8 | 0.445 | 0.00613 | 15 | 6 | 9 |
| trnL(CAA)-ndhB | 584-590 | 590 | 2 | 0.047 | 0.00016 | 2 | 2 | 0 |
| trnL(UAA) intron | 452 | 452 | 8 | 0.616 | 0.00255 | 10 | 2 | 6 |
| trnL(UAA)-trnF(GAA) | 305-363 | 366 | 5 | 0.296 | 0.00258 | 7 | 4 | 3 |
| trnL(UAG)-rpl32 | 545-756 | 792 | 12 | 0.749 | 0.01056 | 51 | 25 | 24 |
| trnM(CAU)-atpE | 214-215 | 216 | 5 | 0.299 | 0.00832 | 9 | 0 | 9 |
| trnN(GUU)-ycf1 | 327 | 327 | 3 | 0.251 | 0.00089 | 2 | 1 | 1 |
| trnP(UGG)-psaJ | 318-319 | 319 | 9 | 0.540 | 0.00439 | 9 | 2 | 7 |
| trnQ(UUG)-psbK | 356-369 | 371 | 7 | 0.339 | 0.00205 | 7 | 3 | 3 |
| trnR(ACG)-trnN(GUU) | 462-470 | 470 | 5 | 0.296 | 0.00127 | 4 | 2 | 2 |
| trnR(UCU)-trnG(UCC) | 212-236 | 252 | 5 | 0.260 | 0.00727 | 13 | 3 | 10 |
| trnS(GCU)-trnC(GCA) | 700-765 | 766 | 10 | 0.718 | 0.00521 | 28 | 10 | 16 |
| trnS(GGA)-rps4 | 319-332 | 332 | 8 | 0.377 | 0.00229 | 9 | 6 | 3 |
| trnS(UGA)-psbZ | 334-347 | 347 | 9 | 0.726 | 0.00634 | 13 | 3 | 9 |
| trnT(GGU)-psbD | 1282-1354 | 1396 | 10 | 0.740 | 0.00466 | 42 | 11 | 30 |
| trnT(UGU)-trnL(UAA) | 539-590 | 598 | 10 | 0.730 | 0.00860 | 28 | 5 | 21 |
| trnV(GAC)-rps7 | 2486-2698 | 2709 | 9 | 0.413 | 0.00034 | 11 | 7 | 14 |
| trnV(GAC)-rrn16S | 226-227 | 227 | 3 | 0.135 | 0.00061 | 2 | 1 | 1 |
| trnV(UAC) intron | 573-577 | 577 | 6 | 0.300 | 0.00281 | 11 | 1 | 10 |
| ycf1-rps15 | 377-391 | 404 | 10 | 0.757 | 0.01063 | 28 | 4 | 24 |
| ycf2-trnL(CAA) | 420 | 420 | 2 | 0.091 | 0.00022 | 1 | 0 | 1 |
| ycf3-trnS(GGA) | 862-946 | 953 | 9 | 0.413 | 0.00272 | 22 | 11 | 11 |
| ycf3 intron 1 | 693-699 | 700 | 11 | 0.515 | 0.00148 | 13 | 6 | 6 |
| ycf3 intron 2 | 738-747 | 748 | 8 | 0.377 | 0.00139 | 14 | 9 | 5 |
| ycf4-cemA | 459-487 | 496 | 10 | 0.571 | 0.00584 | 21 | 5 | 12 |
