# Supplementary material for: Phylogeny of Leontopodium (Asteraceae) in China—with a reference to plastid genome and nuclear ribosomal DNA
Source: Front Plant Sci. 2023 Jul 31;14:1163065. doi: 10.3389/fpls.2023.1163065 (PMC10425225; doi:10.3389/fpls.2023.1163065)
Supplement: Supplementary file 9 [file Table_4.docx]

**Supplementary Table 4 |** Sequence characteristics and nucleotide substitutions models for ML and BI phylogenetic analyses of different datasets (complete cp genomes, coding genes of chloroplast genomes, ITS, ETS, and concatenated sequences of ITS and ETS)

|  | Aligned length (bp) | Variable sites | |  | Parsimony informative sites | | Nucleotide  Diversity (Pi) | Model in ML | Model in BI |
| --- | --- | --- | --- | --- | --- | --- | --- | --- | --- |
|  |  | Numbers | % |  | Numbers | % |  |  |  |
| Complete cp genome | 159,103 | 12,120 | 7.62 |  | 1369 | 0.86 | 0.00521 | TVM+F+I+G4 | GTR+F+I+G4 |
| Coding genes of chloroplast genomes | 74989 | 2126 | 2.84 |  | 455 | 0.61 | 0.00259 | GTR+F+R3 | GTR+F+I |
| ITS | 652 | 128 | 19.63 |  | 74 | 11.35 | 0.03753 | TIM3e+G4 | SYM+G4 |
| ETS | 592 | 132 | 22.30 |  | 79 | 13.34 | 0.04842 | HKY+F+G4 | HKY+F+G4 |
| Concatenated sequences of ITS and ETS | 1244 | 260 | 20.90 |  | 153 | 12.30 | 0.04177 | GTR+F+R3 | GTR+F+G4 |
